# Cobalamin is present in cells of non-tuberculous mycobacteria, but not in *Mycobacterium tuberculosis*

**DOI:** 10.1038/s41598-021-91430-w

**Published:** 2021-06-10

**Authors:** Alina Minias, Filip Gąsior, Anna Brzostek, Tomasz Jagielski, Jarosław Dziadek

**Affiliations:** 1 Laboratory of Genetics and Physiology of Mycobacterium, Institute of Medical Biology, Polish Academy of Sciences, Lodz, Poland; 2 BioMedChem Doctoral School of the University of Lodz and the Institutes of the Polish Academy of Sciences in Lodz, Lodz, Poland; 3 Department of Medical Microbiology, Institute of Microbiology, Faculty of Biology, University of Warsaw, Warsaw, Poland

**Keywords:** Bacterial evolution, Bacterial pathogenesis, Bacterial physiology, Bacterial transcription, Cellular microbiology

## Abstract

Cobalamin (vitamin B12) is a structurally complex molecule that acts as a cofactor for enzymes and regulates gene expression through so-called riboswitches. The existing literature on the vitamin B12 synthesis capacity in *Mycobacterium tuberculosis* is ambiguous, while in non-tuberculous mycobacteria (NTM) is rather marginal. Here we present the results of our investigation into the occurrence of vitamin B12 in mycobacteria. For detection purposes, immunoassay methods were applied to cell lysates of NTM and *M. tuberculosis* clinical and laboratory strains grown under different conditions. We show that whereas vitamin B12 is present in cells of various NTM species, it cannot be evidenced in strains of differently cultured *M. tuberculosis*, even though the genes responsible for vitamin B12 synthesis are actively expressed based on RNA-Seq data. In summary, we conclude that the production of vitamin B12 does occur in mycobacteria, with the likely exception of *M. tuberculosis*. Our results provide direct evidence of vitamin B12 synthesis in a clinically important group of bacteria.

## Introduction

Cobalamin (vitamin B12) is a structurally complex molecule consisting of four linked pyrrole rings and the cobalt ion in the center. There are four chemical forms of cobalamin that differ in the upper ligand: hydroxocobalamin (OHB_12_), methylcobalamin (CH_3_B_12_), deoxyadenosylcobalamin (AdoB_12_), and most chemically stable, cyanocobalamin (CNB_12_).

**Figure 1:**
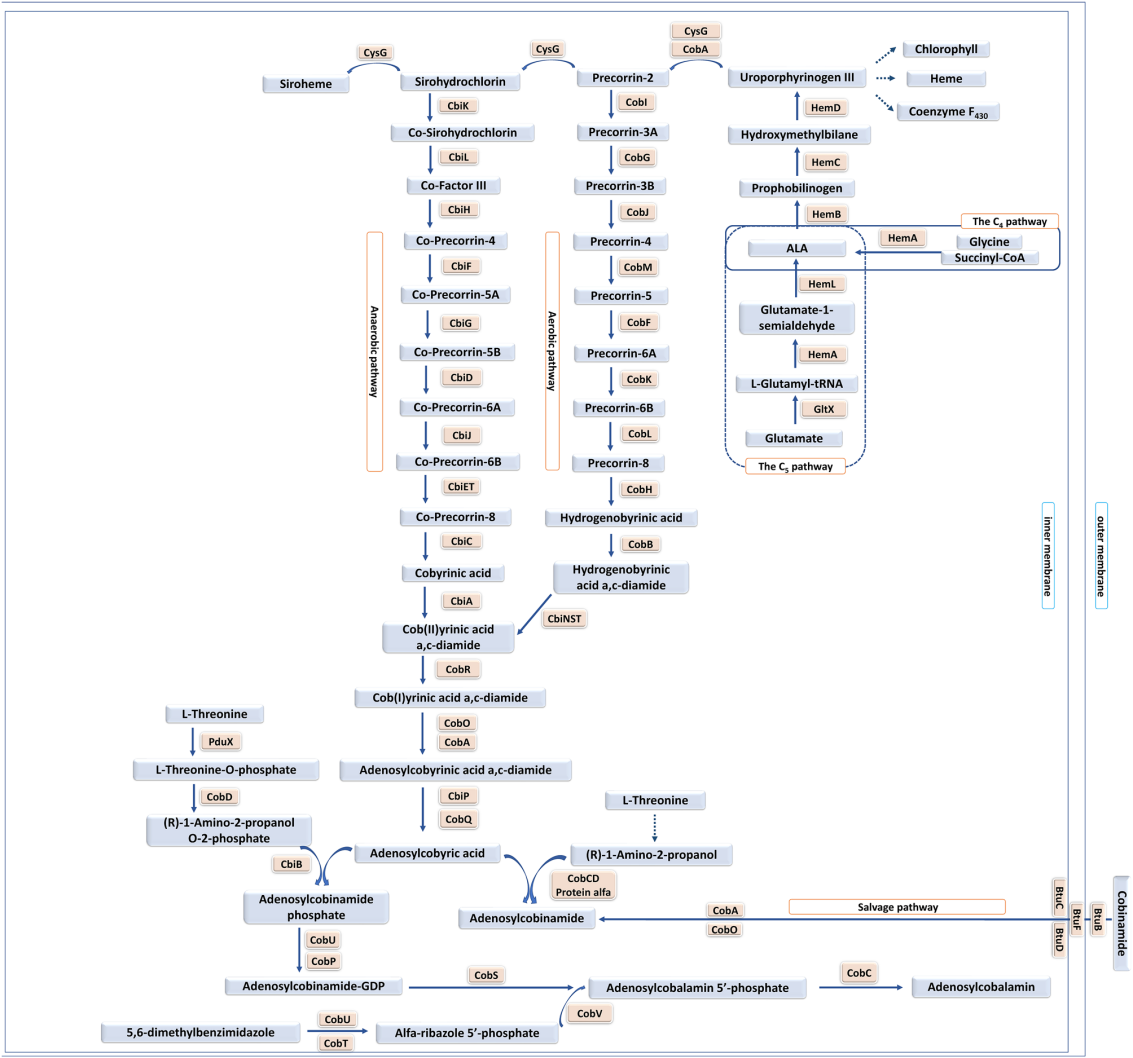
Synthesis of vitamin B12 in bacteria (figure adapted with permission from https://www.ncbi.nlm.nih.gov/pmc/articles/PMC5282855/ under the terms of the Creative Commons Attribution 4.0 International License).

The chemical synthesis of cobalamin involves approximately 70 reactions. Microbial synthesis, which can be aerobic or anaerobic, involves fewer steps (Fig. 1). De novo synthesis involves about 30 reactions starting from glutamate. The salvage pathway is shorter than de novo synthesis, and it involves 12 genes^1, 2^. *Pseudomonas denitrificans*, *Propionibacterium shermanii*, *Sinorhizobium meliloti*, *Eschericha coli* and *Bacillus megaterium* are the main producers of CNB_12_ at the industrial scale^1^. Organisms that use vitamin B12 in their metabolism, and at the same time do not have the gene repertoire enabling its biosynthesis, use exogenous cobalamin actively transported through dedicated ABC transporters^3, 4^.

**Figure 2:**
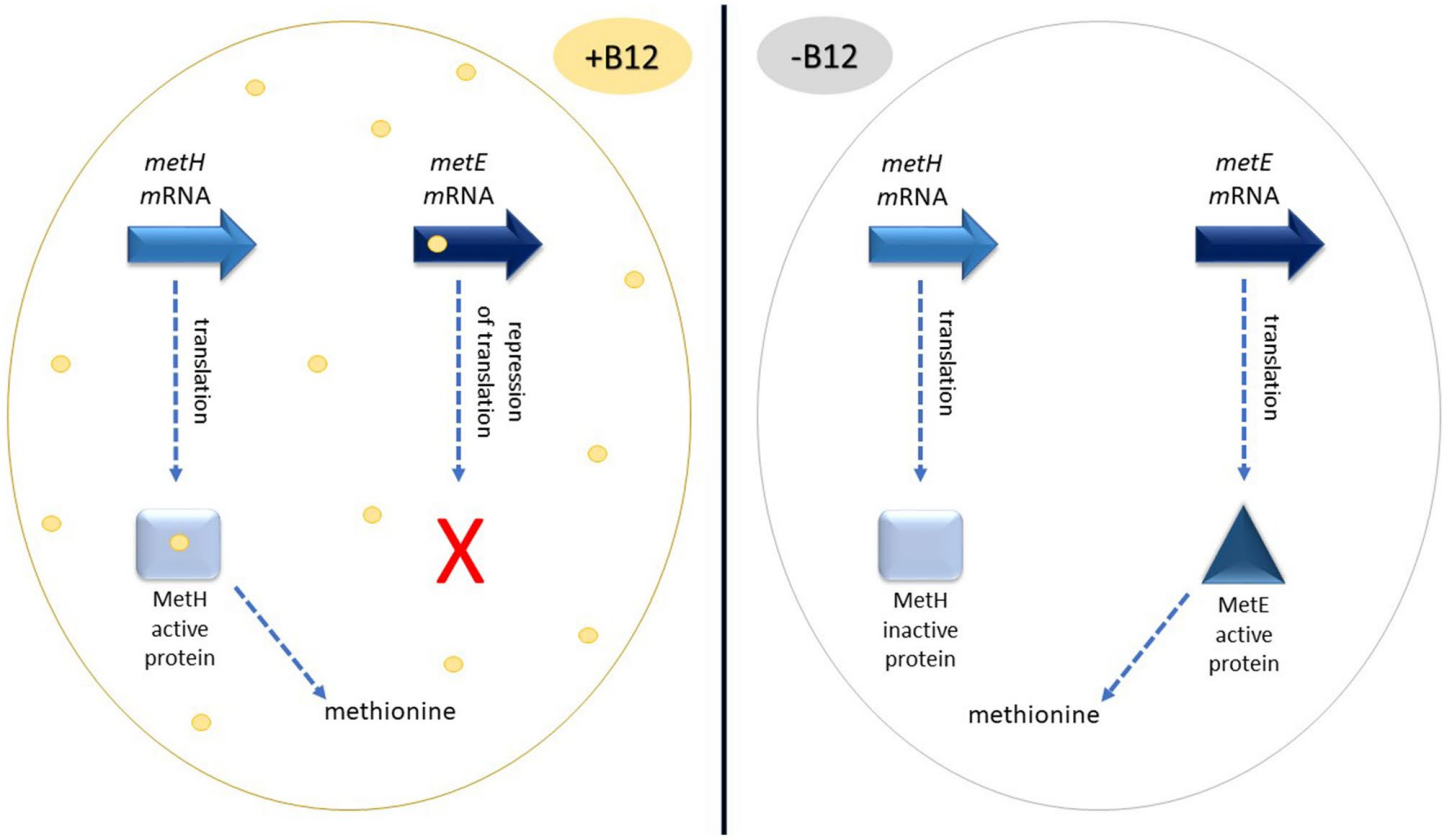
Synthesis of methionine in *M. tuberculosis*. In the presence of vitamin B12 the riboswitch represses the translation of mRNA of *metE*. Cobalamin binds as a cofactor to MetH protein, and the latter provides methionine necessary for the cell. In turn, in the absence of vitamin B12, MetH is not functional. *metE* transcripts are efficiently transcribed to MetE protein, which provides methionine.

Cobalamin influences cell metabolism via two mechanisms. It acts as a cofactor for enzymes, and regulates gene expression through so-called riboswitches. There are three major subfamilies of vitamin B12-dependent enzymes: AdoB_12_-dependent isomerases, CH_3_B_12_-dependent methyltransferases, and dehalogenases. The isomerases are the largest subfamily of B12-dependent enzymes. They play important roles in fermentation pathways. An example of B12-dependent isomerases is methylmalonyl-CoA mutase (MCM), found in bacteria and humans. Together with methylmalonyl-CoA epimerase, the enzyme is involved in converting propionate to succinate through the methylmalonyl-CoA pathway. Here, the enzyme catalyzes the reversible isomerization of l-methylmalonyl-CoA to succinyl-CoA using AdoCbl as a cofactor. Another common B12-dependent isomerase is ribonucleotide reductase (NrdZ). The enzyme catalyzes the conversion of ribonucleotides to deoxyribonucleotides for DNA replication and repair. AdoCbl adenosyl ribose is required to allow hydrogen transfer to the catalytic thiol group^5^. The B12-dependent methyltransferases play an important role in amino acid metabolism and CO_2_ fixation in anaerobic microorganisms. The most extensively studied B12-dependent methyltransferase is methionine synthase (MetH). This enzyme is responsible for the regeneration of methionine from homocysteine via the vitamin B12-dependent pathway and is involved in the folate pathway (Fig. 2). The methyl group of methylcobalamin is transferred to homocysteine forming methionine^6^. Vitamin B12-dependent dehalogenases are present in anaerobic bacteria. Reductive dehalogenases have a vital role in detoxifying aromatic and aliphatic chlorinated organic compounds^7^.

Riboswitches were first detected in *E. coli* (Nou and Kadner, 1998). They are metabolite binding domains in specific mRNAs. Although riboswitches were mostly identified in prokaryotes, they are also present in higher organisms. They react to changes in the environment, such as changes in temperature, pH, or cofactor presence. Ligand binding allows for allosteric rearrangement of the mRNA and results in post-transcriptional control of gene expression. Cobalamin riboswitches repress gene expression by binding the ligand and preventing the mRNA's binding to ribosomes^8^.

The genus *Mycobacterium* accommodates bacterial species that carry genes presumably involved in the synthesis of vitamin B12. Mycobacteria are split into five phylogenetic clades, namely “*tuberculosis-simiae*”, “*terrae*”, “*triviale*”, “*fortuitum-vaccae*”, and “*abscessus-chelonae*”. There are several important pathogens in these groups. The most infamous one is *M. tuberculosis*, a causative agent of tuberculosis. This is an obligatory intracellular human pathogen with a complex life cycle. As shown previously, nearly all genes required for aerobic cobalamin synthesis are identifiable in *M. tuberculosis*, except for the *cobF* coding for precorin-6a synthase^9^.

Further research on *M. tuberculosis* confirmed the presence of two vitamin B12-dependent riboswitches in its genome, encoded by the Rv1133c and Rv0256c genes^10^. Rv1133c encodes a riboswitch regulating the *metE* gene expression of the cobalamin-independent methionine synthase (Fig. 2)^11^. The second cobalamin sensitive riboswitch at Rv0256c affects the PPE2-cobQ1-cobU operon. Rv0256c (PPE2) encodes a PPE2 family protein, while CobQ1 and CobU are presumably involved in vitamin B12 synthesis.

The identification of genes involved in the uptake of vitamin B12 from the environment in *M. tuberculosis* was performed by random mutagenesis^12^. Deletion of the Rv1819c gene, encoding ABC transporter BacA, abolished the ability to transport vitamin B12. Moreover, deletion of the *bacA* did not affect the infectivity of tubercle bacilli, albeit virulence was reduced during prolonged infection^4^.

MetH, MutB, and NrdZ are the three cobalamin-dependent proteins of *M. tuberculosis*. Studies involving these proteins cast doubt whether the reference strain of *M. tuberculosis* H37Rv synthesizes cobalamin. Savvi et al. showed that *M. tuberculosis* H37Rv could not use propionate as a carbon source by using the methylmalonyl pathway without enriching the medium in vitamin B12^13^. Warner et al. showed that B12 supplementation is necessary for the growth of the Δ*metE* mutant of *M. tuberculosis* H37Rv, which also requires the MetH cobalamin-dependent enzyme^11^. Both of these reports suggest that *M. tuberculosis* cannot synthesize cobalamin and relies on cobalamin scavenged from the host. In turn, the *M. tuberculosis* clinical strain CDC1551 was surmised to be able to synthesize cobalamin by demonstrating a truncated variant of MetH. It depends on MetE to synthesize cobalamin. Therefore, it is hyper susceptible to sulfonamides, which block the folate cycle where MetH is involved in the salvage pathway. When *M. tuberculosis* CDC1551 was carrying MetH of H37Rv *in trans*, the authors observed that the strain presented standard susceptibility to sulfonamides. They suspected that intracellular levels of cobalamin in *M. tuberculosis* CDC1551 allow for activation of MetH^14^. In 2018, we showed that genes presumably involved in vitamin B12 synthesis and metabolism are under purifying selective pressure, suggesting functionality of pathway^15^. Ignatov et al. showed that genes involved in vitamin B12 synthesis upregulate their expression during mycobacterial persistence, reached by growing bacteria in a medium deprived of K+^16^. In summary, information regarding the possibility of vitamin B12 synthesis in *M. tuberculosis* is chaotic. As for other mycobacteria, information is scarce. Vitamin B12 synthesis was confirmed in *Mycolicibacterium smegmatis*^17, 18^. One manuscript, published in 1977, currently not available for a full read online, reported the presence of vitamin B12 in the cells of *M. smegmatis*, *Mycolicibacterium fortuitum*, *Mycobacterium asiaticum*, *Mycobacterium phlei*, and *Mycobacterium bovis* BCG using *Lactobacillus leichmannii* ATCC7830 tube method^19^.

Here we present the results of our investigation on the presence of vitamin B12 in mycobacteria. The purpose of this study was to describe vitamin B12 production in phylogenetic order of *Mycobacterium.* We show that vitamin B12 is present in the cells of various non-tuberculous species. Interestingly, we could not identify vitamin B12 in several strains of *M. tuberculosis* cultured under different growth conditions, even though the genes responsible for vitamin B12 synthesis are actively expressed.

## Results and discussion

### Gene expression of vitamin B12 synthesis genes

We aimed to identify the genes involved in vitamin B12 synthesis in NTM included in this study (Table 1). We used whole-genome sequencing data and its annotation found in the major bioinformatics databases. The available data provided an incomplete indication of loci involved in the vitamin B12 biosynthesis pathway, as it is for *M. tuberculosis*. The precision of annotation, covering the entire extent of variability of proteins serving particular functions, is still to be developed.

**Table 1:** Identification of genes involved in vitamin B12 metabolism in various mycobacteria species based on publicly available annotations in major databases.

	Species	*M. tuberculosis H37Rv*	*M. abscessus* subsp*. abscessus*	*M. abscessus* subsp*. bolletii*	*M. conspicuum*	*M. fortuitum*	*M. gastri*	*M. gordonae*	*M. innocens*
	Accession number	NC_000962	NC_010397	CP014950	GCA_010730195	CP011269	LQOX1000000	CP059165	LS999933
	Strain	H37Rv	ATCC 19977	FLAC 003	JCM 14738	CT6	DSM 43505	24T	MK13
	Life cycle	Obligatory pathogen	Opportunistic	Opportunistic	Opportunistic	Opportunistic	Opportunistic	Opportunistic	Opportunistic
	Growth rate	Slow growing	Fast growing	Fast growing	Slow growing	Fast growing	Slow growing	Slow growing	Slow growing
**Gene name**	**Function**								
***Aerobic pathway***									
–	Precorrin-3B methylase, predicted replacement for *cobF*	Rv2067c							
–	Bifunctional protein Rnase H/cobC	Rv2228c							
*cobF*	Precorrin-6A synthase	–	–	–	MCNS_43990	–	AWC07_18155	H0P51_RS23435	EET03_RS22025
*cobA*	Probable cob(I)alamin adenosyltransferase CobO	Rv2849c	–	–	MCNS_15910	XA26_25280	AWC07_16325	H0P51_RS10520	EET03_RS08695
*cobB*	Cobyrinic acid A,C-diamide synthase	Rv2848c	MAB_3155c	–	MCNS_15900	–	AWC07_16320	–	–
*cobC*	L-threonine 3-O-phosphate decarboxylase	Rv2231c	MAB_1902	–	–	–	–	–	–
*cobD*	Adenosylcobinamide-phosphate synthase	Rv2236c	MAB_1898	–	MCNS_32370	–	AWC07_23980	–	–
*cobG*	Precorrin-3B synthase	Rv2064	MAB_2200c	–	MCNS_30550	XA26_35740	AWC07_17760	H0P51_RS16405	EET03_RS14985
*cobH*	Cobalt-precorrin-8 × methylmutase	Rv2065	MAB_2199c	–	MCNS_30560	–	AWC07_17765	–	–
*cobIJ*	Cobalt-precorrin-2 C20-methyltransferase	Rv2066	–	–	MCNS_30570	–	AWC07_17770	–	–
*cobK*	Cobalt-precorrin-6 × reductase	Rv2070c	MAB_2197	–	MCNS_30570	–	AWC07_17795	–	–
*cobL*	Cobalt-precorrin-6y C5-methyltransferase	Rv2072c	MAB_2195	–	MCNS_30630	–	AWC07_17805	–	–
*cobM*	Cobalt-precorrin-4 C11-methyltransferase	Rv2071c	MAB_2196	–	MCNS_30620	XA26_35820	AWC07_17800	H0P51_RS16430	EET03_RS15035
*cobN*	Cobalt chelatase	Rv2062c	MAB_2201	A3N95_10105	MCNS_30500	XA26_35720	AWC07_17750	H0P51_RS16365	EET03_RS14960
*cobO*	Cob(I)alamin adenosyltransferase	Rv2849c	MAB_3156c	–	MCNS_15890	XA26_25260	AWC07_16315	H0P51_RS10510	EET03_RS08685
*cobP*	Adenosylcobinamide kinase/adenosylcobinamide phosphate guanyltransferase	–	–	–	–	–	AWC07_21235	–	–
*cobQ1*	Cobyric acid synthase	Rv0255c	–	A3N95_14850	MCNS_15750	–	–	–	–
*cobQ2*	Putative amidotransferase similar to cobyric acid synthase	Rv3713	MAB_0323c	–	MCNS_50840	–	–	–	–
*cobR*		–	–	–	–	–	–	–	–
*cobS*	Cobalamin synthase	Rv2208	MAB_1952c	–	MCNS_31810	–	AWC07_10855	–	–
*cobT*	Nicotinate-nucleotide–dimethylbenzimidazole phosphoribosyltransferase	Rv2207	MAB_1953c	–	MCNS_31800	XA26_37260	AWC07_10860	H0P51_RS17060	EET03_RS15820
*cobU*	Adenosylcobinamide-phosphate guanylyltransferase	Rv0254c	MAB_1954c	–	MCNS_31790	–	AWC07_21235	–	–
*cobV*		–	–	–	–	–	–	–	–
*pduO*	Cob(I)alamin adenosyltransferase	Rv1314c	–	–	–	XA26_43420	AWC07_13115	–	–
*pduX*		–	–	–	–	–	–	–	–
*bluB*	5,6-Dimethylbenzimidazole synthase	Rv0306	–	–	MCNS_01070	XA26_53750	AWC07_19195	H0P51_RS03095	EET03_RS01545
**Salvage pathway and transport**									
*bacA*	Cobalamin transporter	Rv1819c	–	–	–	–	–	–	–
*btuB*		–		–	–	–	–	–	–
*btuC*	Iron ABC transporter permease	Rv2060	–	–	–	–	–	–	–
*btuD*		–	–	–	–	–	–	–	–
*btuF*		–	–	–	–	–	–	–	–
**Anaerobic pathway**									
*cbiA*	CbiA domain-containing protein	–	–	–	–	–	–	–	–
*cbiB*		–	–	–	–	–	–	–	–
*cbiC*	Precorrin-8X methylmutase	–	–	–	–	–	AWC07_17765	–	–
*cbiD*		–	–	–	–	–	–	–	–
*cbiE*	Precorrin-6Y C(5,15)-methyltransferase	–	–	–	MCNS_30630	–	AWC07_17805	H0P51_RS16435	–
*cbiF*	Precorrin-4 C(11)-methyltransferase	–	–	–	–	–	AWC07_17800	–	–
*cbiG*		–	–	–	–	–	–	–	-
*cbiH*	ATP-binding protein	–	–	–	–	–	AWC07_17770	–	–
*cbiJ*	Cobalt-precorrin-6A reductase	–	–	–	–	–	AWC07_17795	–	–
*cbiK*		–	–	–	–	–	–	–	–
*cbiL*	ATP-binding protein	–	–	–	–	–	AWC07_17770	–	–
*cbiP*		–	–	–	–	–	–	–	–
*cbiT*	Precorrin-6Y-methylase	–	–	–	–	–	AWC07_17805	–	–
*cbiX*	Sirohydrochlorin ferrochelatase	Rv0259c	–	A3N95_07805	–	–	AWC07_14695	–	–
**Urpoporfirynogen III pathway**									
*cysG*	Multifunctional uroporphyrin-III C-methyltransferase/precorrin-2 oxidase/ferrochelatase	Rv2847c	MAB_3143c	–	MCNS_15910	–	AWC07_16325	–	–
*cysH*	Phosphoadenylyl-sulfate reductase	Rv2392	MAB_1661c	–	MCNS_35620	–	AWC07_21315	–	–
*gltX*	Glutamyl-tRNA synthetase	Rv2992c	MAB_3298c	–	–	–	AWC07_23235	–	–
*hemA*	Glutamyl-tRNA reductase	Rv0509	MAB_3993c	–	MCNS_03960	–	AWC07_11800	–	–
*hemB*	Probable delta-aminolevulinic acid dehydratase/porphobilinogen synthase	Rv0512	MAB_3990c	–	MCNS_03990	XA26_52000	–	H0P51_RS04270	EET03_RS02885
*hemC*	Porphobilinogen deaminase	Rv0510	MAB_3992c	–	MCNS_03970	XA26_52020	AWC07_11795	H0P51_RS04260	EET03_RS02875
*hemD*	Uroporphyrinogen III methyltransferase/synthase	Rv0511	–	–	MCNS_03980	–	AWC07_14690	–	–
*hemL*	Glutamate-1-semialdehyde 2,1-aminomutase	Rv0524	–	–	MCNS_04110	XA26_51830	AWC07_11675	H0P51_RS04340	EET03_RS03025
*hemY*	ChlI component of cobalt chelatase	Rv2850c	MAB_2985c	–	MCNS_17540	–	–	–	–
**Vitamin B12 dependent enzymes**									
*metH*	5-Methyltetrahydrofolate–homocysteine methyltransferase	Rv2124c	MAB_2129	–	MCNS_30990	–	AWC07_11205	H0P51_RS04340	EET03_RS15385
*mutB*	Methylmalonyl-CoA mutase	Rv1493	MAB_2711c	–	MCNS_22010	–	–	–	–
*nrdZ*	Ribonucleotide reductase of class II	Rv0570	–	–	–	–	AWC07_08365	–	–

	Species	*M. kansasii*	*M. persicum*	*M. phlei*	*M. porcinum*	*M. terrae*	*M. xenopi*	*M.szulgai*	*M. smegmatis*
	Accession number	GCA_000157895.1	GCA_002705835	GCA_001582015	NZ_MBDY01000007.1	GCA_900187145	NZ_AJFI01000095.1	NZ_LQPW01000016.1	CP000480
	Strain	ATCC 12478	H48	CCUG 21000	ACS 3670	NCTC 10856	RIVM700366	DSM 44166	mc2 155
	Life cycle	Opportunistic	Opportunistic	Opportunistic	Opportunistic	Opportunistic	Opportunistic	Pathogenic	Non-pathogenic
	Growth rate	Slow growing	Slow growing	Fast growing	Fast growing	Slow growing	Slow growing	Slow growing	Fast growing
***Aerobic pathway***									
–	Precorrin-3B methylase, predicted replacement for *cobF*								
–	Bifunctional protein Rnase H/cobC								
*cobF*	Precorrin-6A synthase	MKAN_RS08645	–	–	A5717_31225	–	MXEN_19174	–	MSMEG_5548
*cobA*	Probable cob(I)alamin adenosyltransferase CobO	MKAN_RS09965	CDN37_RS24000	–	A5717_05685	–	MXEN_03569	AWC27_RS04140	
*cobB*	Cobyrinic acid A,C-diamide synthase	–	–	–	A5717_05680	–	MXEN_03564	–	MSMEG_2617
*cobC*	L-threonine 3-O-phosphate decarboxylase	–	–	–	–	–	–	–	–
*cobD*	Adenosylcobinamide-phosphate synthase	MKAN_03275	–	–	A5717_01680	–	MXEN_00720	–	MSMEG_4310
*cobG*	Precorrin-3B synthase	MKAN_RS01775	CDN37_RS01855	MPHLCCUG_RS13200	A5717_31635	–	MXEN_01317	AWC27_RS14575	MSMEG_3871
*cobH*	Cobalt-precorrin-8 × methylmutase	–	–	–	A5717_31640	–	MXEN_01322	–	MSMEG_3872
*cobIJ*	Cobalt-precorrin-2 C20-methyltransferase	–	–	–	A5717_31645	–	MXEN_01327	–	MSMEG_3873
*cobK*	Cobalt-precorrin-6 × reductase	–	–	–	A5717_31665	–	MXEN_01342	–	MSMEG_3875
*cobL*	Cobalt-precorrin-6y C5-methyltransferase	–	–	–	A5717_31675	–	MXEN_01352	–	MSMEG_3878
*cobM*	Cobalt-precorrin-4 C11-methyltransferase	MKAN_RS01815	CDN37_RS01895	MPHLCCUG_RS13225	A5717_31670	–	MXEN_01347	–	MSMEG_3877
*cobN*	Cobalt chelatase	MKAN_RS01760	CDN37_RS01840	MPHLCCUG_RS12530	A5717_31630	–	MXEN_01292	AWC27_RS14585	MSMEG_3864
*cobO*	Cob(I)alamin adenosyltransferase	MKAN_RS23620	CDN37_RS23990	MPHLCCUG_RS15725	A5717_05675	–	MXEN_03569	AWC27_RS04130	MSMEG_2616
*cobP*	Adenosylcobinamide kinase/adenosylcobinamide phosphate guanyltransferase	–	–	–	A5717_01465	–	MXEN_00460	–	–
*cobQ1*	Cobyric acid synthase	–	–	–	–	–	–	–	MSMEG_2588
*cobQ2*	Putative amidotransferase similar to cobyric acid synthase	–	–	–	–	–	MXEN_13996	–	–
*cobR*		*–*	–	–	–	–	–	–	–
*cobS*	Cobalamin synthase	–	–	–	A5717_01475	SAMEA4434518_01622	MXEN_00470	–	MSMEG_4277
*cobT*	Nicotinate-nucleotide–dimethylbenzimidazole phosphoribosyltransferase	MKAN_RS02865	CDN37_RS02735	MPHLCCUG_RS16365	A5717_01470	SAMEA4434518_01623	MXEN_00465	AWC27_RS19945	MSMEG_4275
*cobU*	Adenosylcobinamide-phosphate guanylyltransferase	–	–	–	A5717_01465	SAMEA4434518_00431	MXEN_00460	–	MSMEG_4274
*cobV*		*–*	–	–	–	–	–	–	-
*pduO*	Cob(I)alamin adenosyltransferase	–	–	–	A5717_18045	–	MXEN_16843	–	MSMEG_1544
*pduX*		*–*	–	–	–	–	–	–	-
*bluB*	5,6-Dimethylbenzimidazole synthase	MKAN_RS16250	CDN37_RS16575	MPHLCCUG_RS02360	A5717_14585	–	MXEN_19875	AWC27_RS20945	MSMEG_6053
**Salvage pathway and transport**									
*bacA*	Cobalamin transporter	–	–	–	–	–	–	–	–
*btuB*		*–*	–	–	–	–	–	–	–
*btuC*	Iron ABC transporter permease	–	–	–	A5717_14615	–	MXEN_06686	–	–
*btuD*		*–*	–	–	–	–	–	–	–
*btuF*		*–*	–	–	–	–	–	–	–
**Anaerobic pathway**									
*cbiA*	CbiA domain-containing protein	–	–	–	–	–	MXEN_04563	–	–
*cbiB*		*–*	–	–	–	–	–	–	–
*cbiC*	Precorrin-8X methylmutase	–	–	–	A5717_31640	–	–	–	–
*cbiD*		*–*	–	–	–	–	–	–	–
*cbiE*	Precorrin-6Y C(5,15)-methyltransferase	–	CDN37_RS01900	–	A5717_31675	–	MXEN_01352	AWC27_RS14545	–
*cbiF*	Precorrin-4 C(11)-methyltransferase	–	–	–	A5717_31670	–	MXEN_01347	–	–
*cbiG*		*–*	–	–	–	–	–	–	–
*cbiH*	ATP-binding protein	–	–	–	A5717_31645	–	–	–	–
*cbiJ*	Cobalt-precorrin-6A reductase	–	–	–	A5717_31665	–	MXEN_01342	–	–
*cbiK*		*–*	–	–	–	–	–	–	–
*cbiL*	ATP-binding protein	–	–	–	A5717_31645	–	–	–	–
*cbiP*		*–*	–	–	–	–	–	–	–
*cbiT*	Precorrin-6Y-methylase	–	–	–	A5717_31675	–	MXEN_01352	–	–
*cbiX*	Sirohydrochlorin ferrochelatase	–	-	–	A5717_10190	SAMEA4434518_00227	MXEN_11286	–	–
**Urpoporfirynogen III pathway**									
*cysG*	Multifunctional uroporphyrin-III C-methyltransferase/precorrin-2 oxidase/ferrochelatase	–	–	–	A5717_05685	–	MXEN_03559	–	–
*cysH*	Phosphoadenylyl-sulfate reductase	–	–	–	A5717_28590	SAMEA4434518_01414	MXEN_11291	–	–
*gltX*	Glutamyl-tRNA synthetase	–	–	–	A5717_14355	–	MXEN_16257	–	MSMEG_2383
*hemA*	Glutamyl-tRNA reductase	–	–	–	A5717_22190	SAMEA4434518_00496	MXEN_04673	–	MSMEG_0919
*hemB*	Probable delta-aminolevulinic acid dehydratase/porphobilinogen synthase	MKAN_RS17655	CDN37_RS17885	MPHLCCUG_RS22000	–	SAMEA4434518_00499	–	AWC27_RS21685	MSMEG_0956
*hemC*	Porphobilinogen deaminase	MKAN_RS17645	CDN37_RS17875	MPHLCCUG_RS22010	A5717_22195	SAMEA4434518_00497	MXEN_04668	AWC27_RS07580	MSMEG_0953
*hemD*	Uroporphyrinogen III methyltransferase/synthase	–	-	–	A5717_10195	SAMEA4434518_00498	MXEN_04663	–	MSMEG_0954
*hemL*	Glutamate-1-semialdehyde 2,1-aminomutase	MKAN_RS17800	CDN37_RS18005	MPHLCCUG_RS21920	A5717_22280	SAMEA4434518_00514	MXEN_04593	AWC27_RS07655	MSMEG_0969
*hemY*	ChlI component of cobalt chelatase	–	–	–	–	SAMEA4434518_01694	–	–	–
**Vitamin B12 dependent enzymes**									
*metH*	5-Methyltetrahydrofolate–homocysteine methyltransferase	–	CDN37_RS02225	MPHLCCUG_RS15920	A5717_31970	SAMEA4434518_01721	MXEN_01507	AWC27_RS09650	MSMEG_0093
*mutB*	Methylmalonyl-CoA mutase	–	–	–	–	SAMEA4434518_02142	–	–	MSMEG_3159
*nrdZ*	Ribonucleotide reductase of class II	MKAN_19005	–	–	–	–	MXEN_17528	–	–

We used RNA-Seq data available at ENA Database to estimate gene expression through transcripts per million base pair (TPM) values for genes involved in vitamin B12 synthesis in *M. tuberculosis, M. abscessus* subsp*. abscessus*, and *M. smegmatis* (Table 2). TPM values inform about the level of basal transcription of genes, and are not to be confused with relative gene expression in different conditions. The average gene expression for *M. abscessus* and *M. smegmatis* was 201.88 ± 547.4 TPM and 147.33 ± 607.04 TPM, respectively. In comparison, the average expression of genes predicted to be involved in vitamin B12 synthesis was 94.943 ± 9.483 TPM and 76.669 ± 29.645 TPM, respectively. For *M. tuberculosis* we investigated gene expression level in cells grown in rich broth^20^, in medium supplemented with cholesterol^21^, and in human macrophages^22^. The above conditions' average gene expression was 256.02 ± 551.112 TPM, 256.01 ± 764.53 TPM, and 256.02 ± 1039.71 TPM, respectively. Simultaneously, the average expression of genes predicted to be involved in vitamin B12 aerobic synthesis was lower, 114.114 ± 77.666 TPM, 54.189 ± 35.772 TPM, 145.871 ± 159.664 TPM, respectively. Their overall expression level was comparable to DnaG primase, an essential protein involved in DNA replication (104.986 ± 2.321, 91.056 ± 42.023, and 118.236 ± 98.324, respectively)^23^.

**Table 2:** Expression of genes involved in cobalamin metabolism in *M. tuberculosis* H37Rv based on RNA-Seq data.

Gene name	Description	*M. abscessus* subsp*. abscessus*			*M. smegmatis*			
		Locus	Average	SD	Locus	Average	SD	
All genes			201.88	547.4		147.33	607.04	
**Reference genes**								
sigA	RNA polymerase sigma factor SigA (sigma-A)	MAB_3009	1213.5	31.5941962	–	–	–	
dnaA	Chromosomal replication initiator protein DnaA	MAB_0001	285.34	4.39631664	MSMEG_0093	94.66	39.15167557	
ftsZ	Cell division protein FtsZ	MAB_2009	624.6633333	540.8220485	MSMEG_4222	299.37	47.21376812	
dnaG	Probable DNA primase DnaG	MAB_1708	154.3033333	9.405750014	–	–	–	
rpoB	DNA-directed RNA polymerase (beta chain) RpoB (transcriptase beta chain) (RNA polymerase beta subunit)	MAB_3869c	2151.213333	32.03047351	MSMEG_1367	511.1433333	441.0141973	
**Aerobic pathway**								
–	Precorrin-3B methylase, predicted replacement for cobF	–	–	–	–	–	–	
–	Bifunctional protein Rnase H/cobC	–	–	–	–	–	–	
cobA	Probable cob(I)alamin adenosyltransferase CobO	–	–	–	–	–	–	
cobB	Cobyrinic acid A,C-diamide synthase	MAB_3155c	48.28333333	3.208899084	MSMEG_2617	45.33666667	37.90995428	
cobC	L-threonine 3-O-phosphate decarboxylase	MAB_1902	35.61333333	2.020948622	–	–	–	
cobD	Adenosylcobinamide-phosphate synthase	MAB_1898	30.22333333	2.78363671	MSMEG_4310	34.71333333	5.899494329	
cobG	Precorrin-3B synthase	MAB_2200c	44.81333333	10.51069138	MSMEG_3871	43.05333333	9.251671921	
cobH	Cobalt-precorrin-8 × methylmutase	MAB_2199c	53.07	1.728670009	MSMEG_3872	110.2366667	37.68649802	
cobIJ	Cobalt-precorrin-2 C20-methyltransferase	–	–	–	MSMEG_3873	52.53333333	35.62759651	
cobK	Cobalt-precorrin-6 × reductase	MAB_2197	118.63	8.898027871	MSMEG_3875	40.99	6.827935266	
cobL	Cobalt-precorrin-6y C5-methyltransferase	MAB_2195	95.33	10.65838168	MSMEG_3878	33.24333333	4.282117856	
cobM	Cobalt-precorrin-4 C11-methyltransferase	MAB_2196	190.0066667	34.70278423	MSMEG_3877	40.97333333	4.718308313	
cobN	Cobalt chelatase	MAB_2201	78.53	3.401043957	MSMEG_3864	95.39333333	66.90839808	
cobO	Cob(I)alamin adenosyltransferase	MAB_3156c	94.59	20.00465696	MSMEG_2616	150.34	26.29276136	
cobQ1	Cobyric acid synthase	–	–	–	MSMEG_2588	69.94666667	36.76729162	
cobQ2	Putative amidotransferase similar to cobyric acid synthase	MAB_0323c	79.06333333	7.416537827	–	–	–	
cobS	Cobalamin synthase	MAB_1952c	98.49666667	3.519038694	MSMEG_4277	35.19666667	3.883160225	
cobT	Nicotinate-nucleotide–dimethylbenzimidazole phosphoribosyltransferase	MAB_1953c	171.1133333	8.786639479	MSMEG_4275	34.59333333	4.231930214	
cobU	Adenosylcobinamide-phosphate guanylyltransferase	MAB_1954c	191.44	15.12232456	MSMEG_4274	109.1266667	5.803363967	
pduO	Cob(I)alamin adenosyltransferase	–	–	–	MSMEG_1544	233.6766667	151.377732	
bluB	5,6-Dimethylbenzimidazole synthase	–	–	–	MSMEG_6053	97.35333333	36.85650056	
**Salvage pathway and transport**								
bacA	Cobalamin transporter	–	–	–	–	–	–	
btuC	Iron ABC transporter permease	–	–	–	–	–	–	
**Anaerobic pathway**								
cbiX	Sirohydrochlorin ferrochelatase	–	–	–	–	–	–	
**Urpoporfirynogen III pathway**								
cysG	Multifunctional uroporphyrin-III C-methyltransferase/precorrin-2 oxidase/ferrochelatase	MAB_3143c	95.06666667	7.895709806	–	–	–	
cysH	Phosphoadenylyl-sulfate reductase	MAB_1661c	174.4733333	18.36925783	–	–	–	
gltX	Glutamyl-tRNA synthetase	MAB_3298c	287.6433333	11.14623853	MSMEG_2383	156.9933333	19.13254383	
hemA	Glutamyl-tRNA reductase	MAB_3993c	268.16	8.943438936	MSMEG_0919	174.06	98.84548143	
hemB	Probable delta-aminolevulinic acid dehydratase/porphobilinogen synthase	MAB_3990c	349.51	54.2694122	MSMEG_0956	201.9733333	27.76189895	
hemC	Porphobilinogen deaminase	MAB_3992c	638.4233333	82.95156076	MSMEG_0953	374.95	313.6007022	
hemD	Uroporphyrinogen III methyltransferase/synthase	–	–	–	MSMEG_0954	317.5766667	44.41745753	
hemL	Glutamate-1-semialdehyde 2,1-aminomutase	–	–	–	MSMEG_0969	110.7066667	25.2243619	
hemY	ChlI component of cobalt chelatase	MAB_2985c	196.3366667	1.652099674	–	–	–	
**Vitamin B12 dependent enzymes**								
metH	5-Methyltetrahydrofolate–homocysteine methyltransferase	MAB_2129	605.9966667	39.45958101	MSMEG_0093	94.66	39.15167557	
mutB	Methylmalonyl-CoA mutase	MAB_2711c	180.9466667	2.206928484	–	–	–	
nrdZ	Ribonucleotide reductase of class II	–	–	–	–	–	–	

Gene name	Description	*M. tuberculosis*						
		Locus	Rich 7H9 broth		Cholesterol		Macrophages	
			Average	SD	Average	SD	Average	SD
All genes			256.0163876	551.1120531	256.01	764.53	256.0163722	1039.716894
**Reference genes**								
sigA	RNA polymerase sigma factor SigA (sigma-A)	Rv2703	599.0566667	9.565264706	630.73	106.0339119	1776.92	403.8168363
dnaA	Chromosomal replication initiator protein DnaA	Rv0001	131.0233333	1.802615384	532.06	40.00305738	579.74	351.8220045
ftsZ	Cell division protein FtsZ	Rv2150c	691.0366667	72.64343069	200.9733333	88.35943501	548.8	187.4937259
dnaG	Probable DNA primase DnaG	Rv2343c	104.9866667	2.321354968	91.05666667	42.02332077	118.2366667	98.34518335
rpoB	DNA-directed RNA polymerase (beta chain) RpoB (transcriptase beta chain) (RNA polymerase beta subunit)	Rv0667	1650.64	35.26432853	701.45	81.97233192	438.5966667	130.3168532
**Aerobic pathway**								
–	Precorrin-3B methylase, predicted replacement for cobF	Rv2067c	104.4866667	4.4877339	32.90666667	33.05350242	283.67	60.07031935
–	Bifunctional protein Rnase H/cobC	Rv2228c	91.63333333	0.241430919	36.25333333	20.75320752	0	0
cobA	Probable cob(I)alamin adenosyltransferase CobO	Rv2849c	77.32666667	3.099519676	74.58333333	10.26311302	507.9333333	458.6724143
cobB	Cobyrinic acid A,C-diamide synthase	Rv2848c	51.04333333	0.651783877	60.56666667	31.54769652	0	0
cobC	L-threonine 3-O-phosphate decarboxylase	Rv2231c	78.29	3.854279007	37.80333333	27.56415224	0	0
cobD	Adenosylcobinamide-phosphate synthase	Rv2236c	58.52333333	1.689108904	1.9	1.064831755	81.79333333	115.6732413
cobG	Precorrin-3B synthase	Rv2064	183.29	4.164956983	33.45	26.14527236	133.5466667	188.8635072
cobH	Cobalt-precorrin-8 × methylmutase	Rv2065	178.23	4.38254112	0.593333333	0.839100047	0	0
cobIJ	Cobalt-precorrin-2 C20-methyltransferase	Rv2066	150.18	9.995749096	74.32666667	8.916786915	72.98	58.51295412
cobK	Cobalt-precorrin-6 × reductase	Rv2070c	89.46666667	8.680419857	42.11333333	30.69800681	419.98	124.7825583
cobL	Cobalt-precorrin-6y C5-methyltransferase	Rv2072c	51.73666667	2.388616522	56.36333333	44.1996458	219.44	310.3350241
cobM	Cobalt-precorrin-4 C11-methyltransferase	Rv2071c	32.80333333	1.224100577	63.16666667	39.84209192	0	0
cobN	Cobalt chelatase	Rv2062c	101.4866667	3.59177146	46.24666667	13.10098554	167.3266667	118.4526528
cobO	Cob(I)alamin adenosyltransferase	Rv2849c	77.32666667	3.099519676	74.58333333	10.26311302	507.9333333	458.6724143
cobQ1	Cobyric acid synthase	Rv0255c	83.46333333	4.16039528	60.76333333	17.12575124	0	0
cobQ2	Putative amidotransferase similar to cobyric acid synthase	Rv3713	178.4833333	3.066206487	95.84333333	45.96082849	104.7666667	148.1624409
cobS	Cobalamin synthase	Rv2208	280.7333333	5.75070044	38.15	35.52983347	228.8033333	323.5767771
cobT	Nicotinate-nucleotide–dimethylbenzimidazole phosphoribosyltransferase	Rv2207	341.1866667	6.934096112	10.67	6.638649461	70.94666667	100.3337382
cobU	Adenosylcobinamide-phosphate guanylyltransferase	Rv0254c	46.93666667	5.12625486	171.9133333	89.37495001	138.89	196.4201217
pduO	Cob(I)alamin adenosyltransferase	Rv1314c	88.84	1.75789647	51.02	33.75337119	125.2866667	177.1821032
bluB	5,6-Dimethylbenzimidazole synthase	Rv0306	50.93	3.946044433	74.76	42.37912772	0	0
**Salvage pathway and transport**								
bacA	Cobalamin transporter	Rv1819c	112.5766667	3.598373089	27.63	21.66365312	82.66333333	59.02668455
btuC	Iron ABC transporter permease	Rv2060	191.76	5.638421765	296.6266667	162.4777085	106.7166667	150.9201573
**Anaerobic pathway**								
cbiX	Sirohydrochlorin ferrochelatase	Rv0259c	12.73	1.498465882	0	0	0	0
**Urpoporfirynogen III pathway**								
cysG	Multifunctional uroporphyrin-III C-methyltransferase/precorrin-2 oxidase/ferrochelatase	Rv2847c	68.92	5.318890862	36.77	10.84717782	63.26	89.46314996
cysH	Phosphoadenylyl-sulfate reductase	Rv2392	312.9333333	19.43125032	503.0566667	166.2576146	0	0
gltX	Glutamyl-tRNA synthetase	Rv2992c	170.96	11.21727537	89.43333333	42.90810128	107.7533333	76.94248949
hemA	Glutamyl-tRNA reductase	Rv0509	612.7766667	65.26401271	698.94	222.6659401	362.77	513.034254
hemB	Probable delta-aminolevulinic acid dehydratase/porphobilinogen synthase	Rv0512	279.2466667	19.95736511	165.0033333	36.41366075	160.32	114.4782576
hemC	Porphobilinogen deaminase	Rv0510	627.0233333	64.43651415	435.5433333	115.7868324	161.2566667	114.1554689
hemD	Uroporphyrinogen III methyltransferase/synthase	Rv0511	640.4733333	56.28158037	342.2533333	64.04123949	138.85	9.488702054
hemL	Glutamate-1-semialdehyde 2,1-aminomutase	Rv0524	437.8066667	25.11909809	114.9666667	72.38097832	391.8	135.6539762
hemY	ChlI component of cobalt chelatase	Rv2850c	135.66	6.78823001	38.01666667	3.454488224	81.53333333	115.3055458
**Vitamin B12 dependent enzymes**								
metH	5-Methyltetrahydrofolate–homocysteine methyltransferase	Rv2124c	155.78	9.823003614	311.4966667	16.31473431	128.15	45.50090622
mutB	Methylmalonyl-CoA mutase	Rv1493	48.83	1.851323851	139.7433333	70.5155852	38.08333333	53.8579665
nrdZ	Ribonucleotide reductase of class II	Rv0570	60.79333333	1.360890232	366.2433333	120.7928051	119.6	101.265619

Studies with *Propionibacterium* sp. showed the crucial role of *cobA* gene in regulating the level of synthesis of vitamin B12. Vitamin B12 was shown to regulate the *cobA* operon through a riboswitch in its 5′ untranslated region (5′ UTR)^24^. Similarly, *M. tuberculosis* contains a PPE2-*cobQ1*-*cobU* operon, containing vitamin B12 synthesis genes and controlled by a riboswitch. Taken the ubiquity of vitamin B12 riboswitches across Prokarytotes, the mechanisms where the level of vitamin B12 synthesis genes seem to be controlled by the synthesis product might be common^25^. Presented results show that *cobQ1* and *cobU* of *M. tuberculosis* are actively expressed in a rich broth and in the presence of cholesterol. Expression of *cobQ1* was not observed in macrophages. The level of reading coverage of the mycobacterial genome is relatively low. We assume that the low coverage results from natural technical difficulties of isolating mycobacterial RNA from the Eukaryotic cells that have RNA of their own^26^. Since reads of *cobU* are present, we suspect that the absence of *cobQ1* reads in macrophages is due to too low coverage.

### Vitamin B12 concentration in non-tuberculous mycobacteria

**Figure 3:**
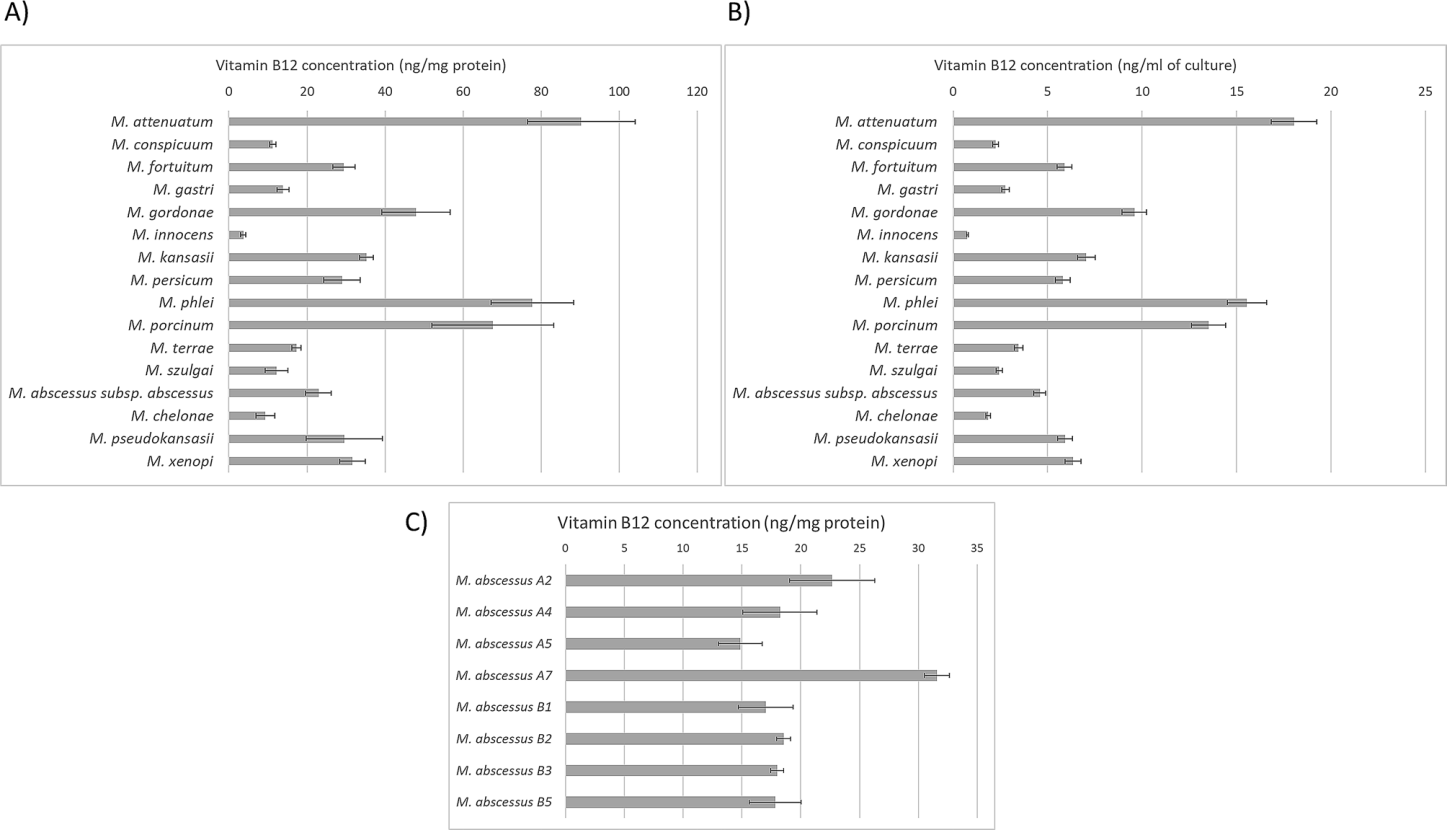
Cobalamin concentration in cell lysates of non-tuberculous mycobacteria. Cobalamin was detected in cell lysates of non-tuberculous mycobacteria by immunoassay. Cells were grown in 7H9 medium supplemented with OADC, Tween 80, and CoCl_2._ We cultured cells until the suspension reached OD_600_ = 1. Next, cells were harvested and washed with fresh medium without supplements to remove residual medium proteins from the surface. The pellet was re-suspended in Tris buffer and disrupted by beat-beating. The suspension was spinned. We used supernatant to estimate the concentration of vitamin B12 and protein content. Results were obtained from three independent cultures; each lysate was analyzed in two technical replicates. (**A**) cobalamin concentration in cell lysates of non-tuberculous mycobacteria, when normalized in ng per mg of protein, (**B**) cobalamin concentration in non-tuberculous mycobacteria cell lysates, when normalized in ng per ml of culture, (**C**) cobalamin concentration in cell lysates of clinical strains of *M. abscessus* complex. Whiskers represent SD.

We measured the concentration of vitamin B12 per mg of protein in cell lysates obtained from 7H9 medium cultures of various species of NTM spread across the phylogenetic tree (Fig. 3A)^27^. On average, mycobacterial cells contained 33.044 ng of cobalamin per mg of protein. The median level of vitamin B12 across analyzed cells was 29.217 ng per mg of protein. The lowest concentration of vitamin B12 was detected for *M. innocens* (3.704 ± 0.643 ng/mg of protein). The highest concentration of vitamin B12 was detected in *M. attenuatum* (90.211 ± 13,769 ng/mg of protein). Results regarding relatively high production of vitamin B12 in *M. phlei* (77.712 ± 10.597 ng/mg of protein), when compared with other species of mycobacteria, are in line with previous findings from 1977^19^. When vitamin B12 concentration was normalized to protein content, we detected a higher concentration of vitamin B12 in mycobacteria than it was previously detected in *P. aeruginosa*. There, analyses by HPLC–MS detected from 0.32 to 3.72 ng of vitamin B12 per mg of protein, depending on culture conditions and strain^28^.

There are different approaches to the normalization of vitamin B12 concentration in bacteria. To further compare our results with other bacterial species, we also normalized our data regarding vitamin B12 concentration to ml of culture (Fig. 3B). When calculated in such a way, we obtained from 0.049 to 1.2 ng of vitamin B12 per ml of culture. In comparison, *Pseudomonas freudenreichii* produced from 20 to 125 ng of vitamin B12/ ml of culture, depending on culture conditions^29^. *B. megaterium* produced from 0.26 ng/ml of culture to 204 ng/ml of culture, also depending on the culture conditions. Due to relatively low concentration of vitamin B12, expensive growth media, long culture time, and difficulties to disrupt the cells, we conclude that mycobacteria are not attractive alternative producers of vitamin B12 at the industrial scale.

Importantly, we show that NTM can produce vitamin B12, and synthesis is shared across the phylogenetic tree. The sensitivity of the immunoassay detection was suitable for the detection of vitamin B12 in mycobacterial cells. This observation is an important reference point for results obtained for *M. tuberculosis*.

### The level of vitamin B12 concentration in the NTM cells is variable, and it depends on the cell line

*M. abscessus* complex is a group of non-tuberculous mycobacteria. It is an emerging human pathogen often associated with the infection of cystic fibrosis patients. It consists of three subspecies *M. abscessus* subsp. *abscessus*, *M. abscessus* subsp. *massiliense* and *M. abscessus* subsp. *bolletii*. We measured the concentration of vitamin B12 per mg of protein in cell lysates obtained from 7H9 medium cultures of various clinical strains of *M. abscessus* subsp. *abscessus* and *M. abscessus* subsp. *bolletii* (Fig. 3C). We detected vitamin B12 in cells of all of the analyzed strains. On average, cells contained 19.842 ng of cobalamin per mg of protein. The median level of vitamin B12 across analyzed cells was 18.121 ng per mg of protein. The lowest concentration of vitamin B12 was detected for *M. abscessus* subsp. *abscessus* strain A5 (14.861 ± 1.848). The highest concentration of vitamin B12 was detected in *M. abscessus* subsp. *abscessus* strain A7 (31.582 ± 1.071). The difference in concentration between the highest and the lowest producing strain was statistically significant (*p* < 0.01, t = 11.07, df = 3).

We observed up to a twofold difference in the level of vitamin B12 synthesis across distinct strains of the same species. Strain variability in cobalamin concentration was observed previously in *Pseudomonas aeruginosa*, where the concentration of vitamin B12 ranged from 0.84 to 3.72, hence changed four-fold, depending on a strain^28^. Possible sources of the variability in the production of vitamin B12 in different strains are mutations either in the promoter regions of genes involved in the synthesis or directly in coding sequences, resulting in enzymes with altered reaction rates^30^.

Strain variability in the level of vitamin B12 production is important in the context of *M. tuberculosis*. Data presented in previous manuscripts suggested indirectly that certain strains of *M. tuberculosis* may be capable of cobalamin synthesis, while others are probably not^14, 31^. As in other species, mycobacteria do show a certain spread in the level of vitamin B12 synthesis that probably can be attributed to the genetic background rather than the environmental factors or stage of the growth.

### Increased concentration of vitamin B12 in mycobacterial cells under starvation results from accumulation rather than increased production

In our previous study, we showed that cells of *M. smegmatis* grown in a medium deprived of nutrients contain an approximately eightfold amount of vitamin B12 when compared to cultures grown in a rich broth. An increase in vitamin B12 concentration was also observed in stationary phase cultures^17^. A similar observation was made in *P. aeruginosa*. There, vitamin B12 concentration increased from non-detectable during exponential growth to 0.32–0.67 ng/mg of protein in stationary phase cultures, depending on a strain. The concentration further increased up to 3.72 in conditions of continuous-flow growth^28^.

**Figure 4:**
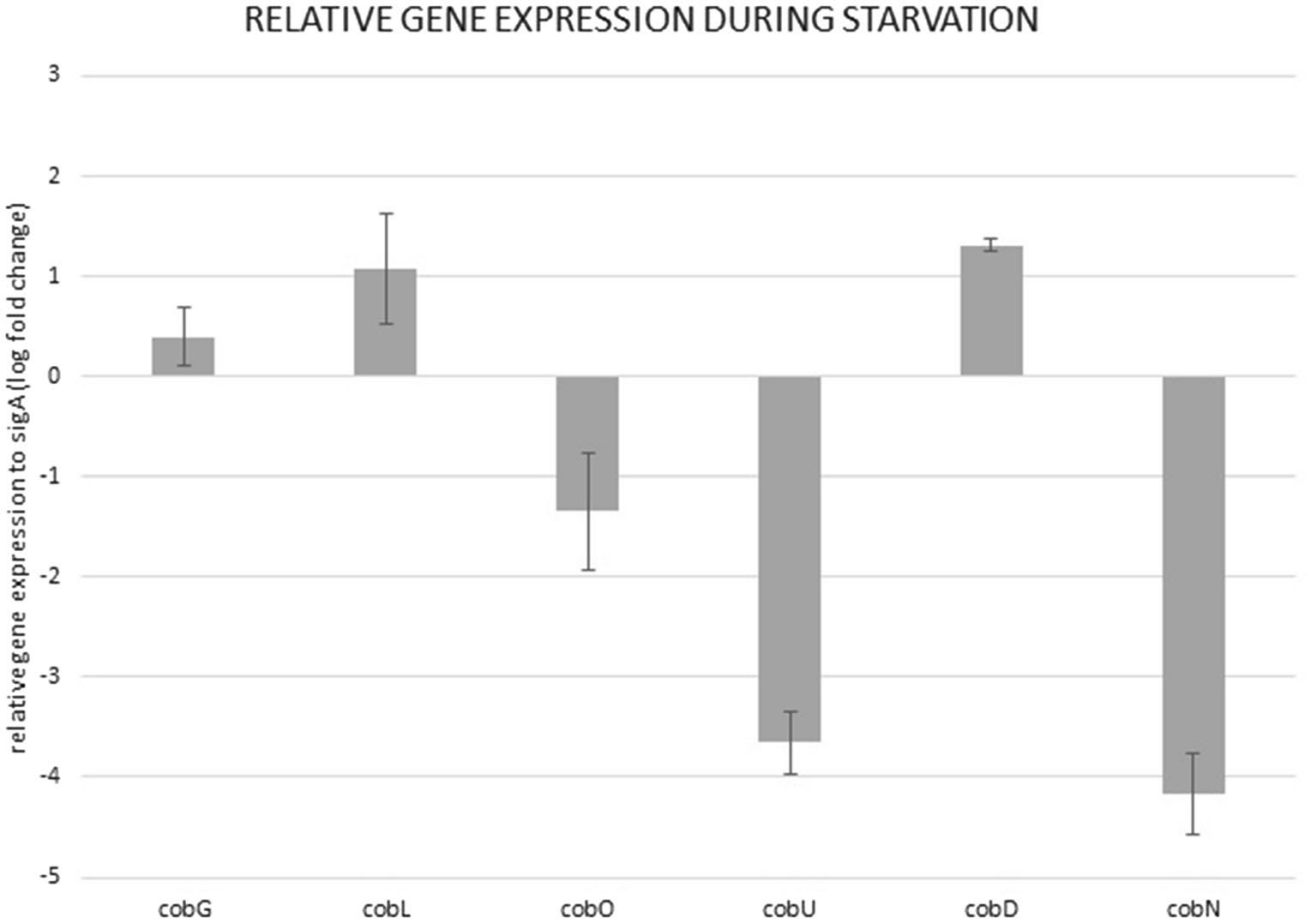
Relative gene expression of genes involved in cobalamin biosynthesis in *M. smegmatis*. Data across samples was normalized to *sigA*. Bars represent log fold change. Whiskers represent SD.

Here, we show that the reason behind the increased concentration of vitamin B12 in starved cells of *M. smegmatis* mc^2^ probably results from accumulation rather than increased synthesis. We estimated the relative gene expression of genes involved in cobalamin synthesis in starved cells compared to cells in the logarithmic phase (Fig. 4). We observed that the expression of genes involved in vitamin B12 synthesis was either constitutive (0 to 1-fold change in relative expression to *sigA*) for *cobG*, *cobL*, cobO and *cobD* or repressed (> 3-fold change) for *cobU* and *cobN*.

Accumulation of vitamin B12 in starved cells and old cultures of *M. smegmatis* is important in the context of cobalamin detection in *M. tuberculosis*. *M. smegmatis* is a model organism for studying the biology of mycobacteria, including M. *tuberculosis*^32^. Bacteria of the same phylogenetic order are likely to maintain the same biological pathways and mechanisms. Indeed, increased expression of cobalamin synthesis genes was reported in dormant cultures of *M. tuberculosis*^16^. Therefore, if cobalamin was to be present in the cells of *M. tuberculosis*, it was more likely to be identified in prolonged, starved, or dormant cultures.

### Lack of observable vitamin B12 production in *M. tuberculosis*

We tested the contents of cells of *M. tuberculosis* for vitamin B12 by immunoassay (Table 3). We included laboratory strain of *M. tuberculosis* H37Rv and five clinical strains of *M. tuberculosis,* here grouped into a group of “clinical strains”. As a negative control strain, we used *M. tuberculosis* deficient in *cobIJ* gene. Predicted function of *cobIJ* is precorrin-2 C20-methyltransferase/precorrin-3B C17-methyltransferase. The gene product is required at the early stage of vitamin B12 synthesis (Fig. 1).

**Table 3:** Types of cultures tested for cobalamin in cells of *M. tuberculosis* H37Rv, five clinical strains of *M. tuberculosis,* and ∆*cobIJ*.

Type of culture	Growth medium	Supplementation	Vitamin B12
Logarytmic phase cultures	7H9 + OADC + Tween 80 + CoCl2	–	Negative
Stationary phase cultures	7H9 + OADC + Tween 80 + CoCl2	–	Negative
Acidified cultures	7H9 + OADC + Tween 80 + CoCl2, pH 5.5	–	Negative
Starved cultures	7H9 + Tween 80 + CoCl2	–	Negative
Persister cultures	K + deficient Sauton medium + CoCl2	–	Negative
Hypoxic cultures	7H9 + OAD + Tween 80 + CoCl2 (+ methylene blue), 25% head ratio	–	Negative
Logarytmic phase cultures	7H9 + OADC + Tween 80 + CoCl2	Uroporphyrinogen III	Negative
Stationary phase cultures	7H9 + OADC + Tween 80 + CoCl2	Uroporphyrinogen III	Negative
Persister cultures	K + deficient Sauton medium + CoCl2	Uroporphyrinogen III	Negative
Hypoxic cultures	7H9 + OAD + Tween 80 + CoCl2 (+ methylene blue), 25% head ratio	Uroporphyrinogen III	Negative

We screened *M. tuberculosis* cell lysates derived from cultures grown in various conditions. The growth conditions aimed to mimic the environments that can be found during *M. tuberculosis* infection cycle. All cultures were supplemented with cobalt to evade the blockade of synthesis due to insufficient cobalt concentration. First, we investigated the possibility of de novo synthesis of vitamin B12. We tested logarithmic phase cultures, stationary phase cultures, and acidified cultures that would mimic the infection's active stage. For granuloma conditions, we tested starved cultures, persister cultures, and hypoxic cultures. ELISA immunoassay detected less than one ng of vitamin B12 per one ml of lysate in all of the samples. The samples were considered negative for vitamin B12 based on the cut-off value of the sensitivity of the test. Taken that vitamin B12 tends to accumulate in the cells during prolonged growth, our results suggest that it is unlikely that there is an ongoing de novo synthesis of vitamin B12 inside *M. tuberculosis* cells.

Next, we wanted to see if *M. tuberculosis* might rely on substances widely present in the host to produce vitamin B12. We supplemented the growth medium with uroporphyrinogen III, which is a precursor of heme in the human body and a precursor of vitamin B12 in bacteria (Fig. 1). We tested cell lysates from logarithmic phase cultures, stationary phase cultures, persister cell cultures, and hypoxic cultures. ELISA immunoassay detected less than one ng of vitamin B12 per one ml of lysate in all of the samples. Hence, the samples were considered negative for vitamin B12 based on the cut-off value of the test's sensitivity.

### *M. tuberculosis metE* promoter responds to vitamin B12 concentration present in the host

**Figure 5:**
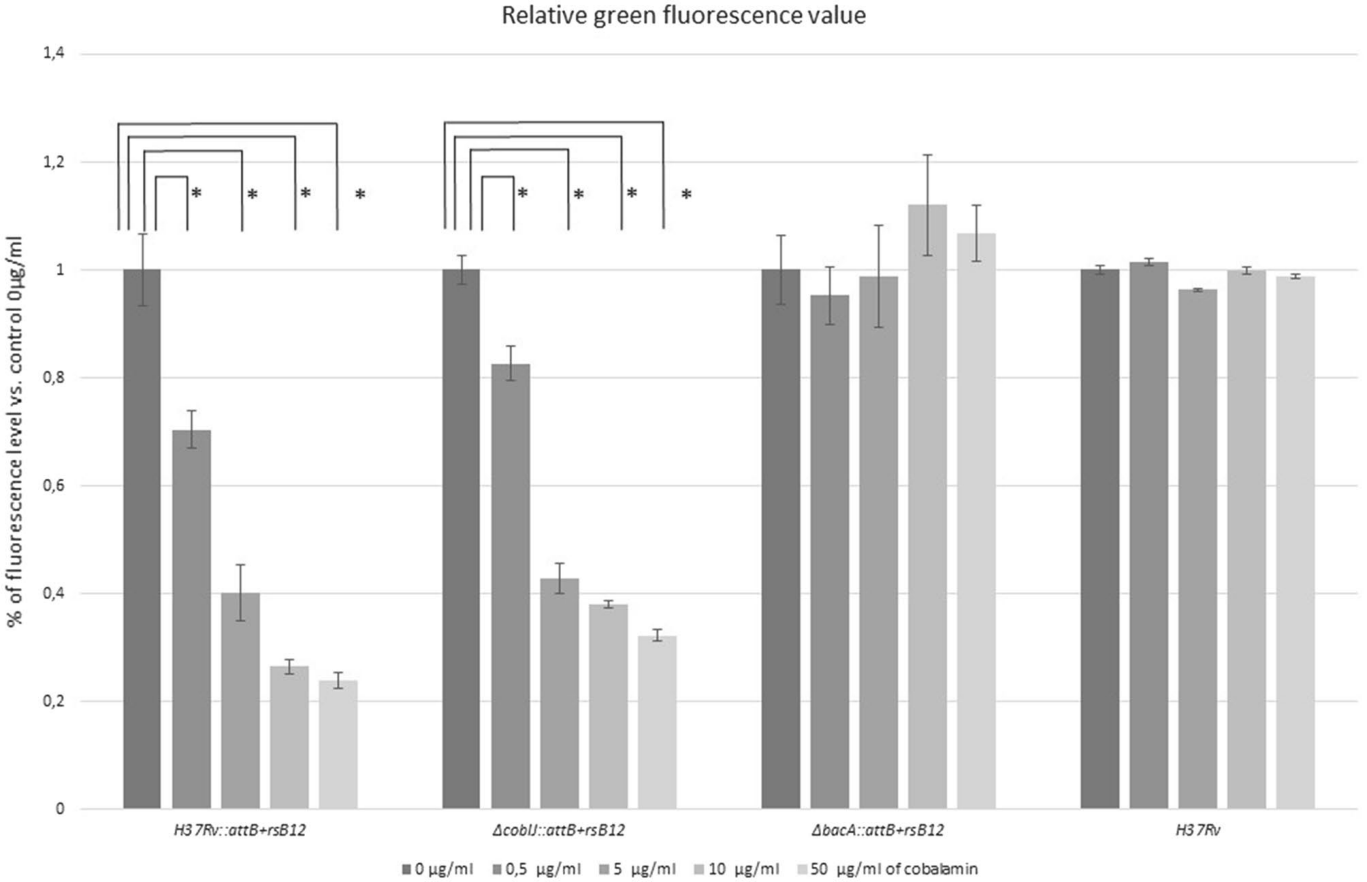
The evaluation of the protein expression of *gfp* gene under the control of vitamin B12- dependent riboswitch, based on the green fluorescence. We used a Guava flow cytometer to evaluate the green fluorescence in cells of H37Rv::*attB* + rsB12, ∆*bacA*::*attB* + rsB12, ∆*cobIJ*::*attB* + rsB12, carrying the gene of GFP under the control of the *metE* promoter, controlled by a vitamin B12-dependent riboswitch. We observed that the green fluorescence of strains H37Rv::*attB* + rsB12 and ∆*cobIJ*::*attB* + rsB12 significantly differed from the control strains grown in medium without exogenous vitamin B12 supplementation. Each strain was analyzed in samples collected from three cultures. Statistical analysis was performed with paired t-test. Whiskers represent SD.

We used the GFP reporter system to see if the cells of *M. tuberculosis* could respond to vitamin B12 concentration found within the host (Fig. 5). Vitamin B12 concentration in the human body is between 0.2 and 0.9 μg/ml. We constructed a series of mutants, H37Rv::*attB* + rsB12, ∆*bacA*::*attB* + rsB12, ∆*cobIJ*::*attB* + rsB12, carrying the gene of GFP under the control of the *metE* promoter, controlled by a vitamin B12-dependent riboswitch. In our model, the presence of vitamin B12 in the cells prevents translation of *gfp* transcript, which results in diminished fluorescence. Of note, distinct clones of the same cell lines showed a different level of basal fluorescence without supplementation of medium without vitamin B12. Therefore, the fluorescence level could not be reliably compared between different cell lines due to the distinct basal expression of GFP in the clones. However, green fluorescence levels could be relatively compared within one clone of the cell line when considering different concentrations of vitamin B12 in the growth medium. We tested various concentrations of vitamin B12. We observed that supplementation of the growth medium with vitamin B12 gradually diminished gene expression of the green fluorescence protein of *M. tuberculosis* H37Rv and ∆*cobIJ* from 100 to 23.93% and 23.70%, respectively. In turn, the green fluorescence expression of ∆*bacA* was not affected, and it remained constant at approximately 100%. Similarly, the autofluorescence level was constant for the control strain *M. tuberculosis* H37Rv, which lacked the reporter system. *M. tuberculosis* H37Rv GFP expression diminished to 70.29% in the presence of 0.5 μg/ml of vitamin B12 (*p* = 0.01, t = 6.64, df = 3). Hence, *M. tuberculosis metE* promoter is responsive to vitamin B12 concentration found in the human body. Our results confirm the role of BacA as the transporter of vitamin B12^33^. Further, our results indirectly confirm the lack of vitamin B12 production in *M. tuberculosis* H37Rv, because the wild type strain and the knock-out strain similarly showed a decrease in fluorescence corresponding to increasing concentration of supplemented vitamin B12.

Previous reports suggested that the ability to synthesize vitamin B12 by *M. tuberculosis* was restricted in *M. cannetti* like ancestor^9, 10^. *M. tuberculosis* is an obligate pathogen with possible access to vitamin B12 from the host. It is, therefore, possible that the genes involved in vitamin B12 synthesis in the genome of *M. tuberculosis* are remnants from a more independent ancestor. The genes of the vitamin B12 biosynthesis pathway in *Mycobacterium leprae*, another obligate pathogen of *Mycobacterium* genus, evolved into pseudogenes^10^. The most probable explanation is that *M. tuberculosis* does not synthesize vitamin B12 anymore. However, there was still not enough time since the abrogation of the pathway to accumulate mutations that would entirely degrade the pathway, impede the expression of genes and convert them into pseudogenes. It seems that the disruption of the pathway might have taken place relatively recently, as *cobF* encoding region was found in two *M. tuberculosis* strains found in the African Great Lakes region, representing Lineage 8 of *M. tuberculosis* complex^31^. To be precise, it cannot be excluded that *M. tuberculosis* does synthesize vitamin B12, but the level of vitamin concentration is undetectable by the immunoassay we used. Finally, it remains to be established whether *M. tuberculosis* is able to synthesize cobamides other than vitamin B12^34^.

## Summary

We conclude that mycobacteria are generally capable of vitamin B12 synthesis, with the likely exception of *M. tuberculosis*. Our results are direct evidence of vitamin B12 production in these clinically important group of bacteria.

## Materials and methods

### Bacterial strains

We analyzed the level of vitamin B12 in several type strains and clinical strains of non-tuberculous mycobacteria (Table 4). *Mycolicibacterium porcinum*, *M. fortuitum*, and *Mycobacteroides abscessus* complex were isolated and differentiated in Canada^35^. Further, we included a laboratory strain of *M. tuberculosis* H37Rv, its genetically modified derivatives Δ*cobIJ* and Δ*bacA*, and five clinical strains of *M. tuberculosis* isolated in Lodz, Poland, between 2006 and 2008. Each strain belonged to a different clade. We chose strains: 321 (spoligotype 35, clade H4), 404 (spoligotype 46, clade U (likely H)), 663 (spoligotype 50, clade H3), 216/8 (spoligotype 42, clade LAM9) and 218/8 (spoligotype 1253, clade S)^36^. We used *Escherichia coli* Top10 for cloning.

**Table 4:** List of strains used in this study.

Species	Strain	Description
**Non-tuberculous mycobacteria**		
*M. smegmatis*	mc2	Type strain
*M. attenuatum*	DSM 107153	Type strain
*M. chelonae*	ATCC 35752	Type strain
*M. conspicuum*	DSM 44136	Type strain
*M. gastrii*	DSM 43505	Type strain
*M. innocens*	DSM 107161	Type strain
*M. kansasii*	ATCC12478	Type strain
*M. persicum*	DSM 104278	Type strain
*M. phlei*	JCM 5865	Type strain
*M. pseudokansasii*	DSM 107152	Type strain
*M. szulgai*	DSM 44166	Type strain
*M. terrae*	JCM 12143	Type strain
*M. fortuitum*	F1	Clinical isolate
*M. gordonae*	G1	Clinical isolate
*M. porcinum*	P1	Clinical isolate
*M. xenopi*	X1	Clinical isolate
*M. abscessus* subsp*. abscessus*	A2	Clinical isolate
*M. abscessus* subsp*. abscessus*	A4	Clinical isolate
*M. abscessus* subsp*. abscessus*	A5	Clinical isolate
*M. abscessus* subsp*. abscessus*	A7	Clinical isolate
*M. abscessus* subsp*. bolletii*	B1	Clinical isolate
*M. abscessus* subsp*. bolletii*	B2	Clinical isolate
*M. abscessus* subsp*. bolletii*	B3	Clinical isolate
*M. abscessus* subsp*. bolletii*	B5	Clinical isolate
***M. tuberculosis***		
*M. tuberculosis*	H37Rv	Type strain, wild type
*M. tuberculosis*	321	Clinical isolate
*M. tuberculosis*	404	Clinical isolate
*M. tuberculosis*	663	Clinical isolate
*M. tuberculosis*	216/8	Clinical isolate
*M. tuberculosis*	218/8	Clinical isolate
**Genetically modified strains**		
*M. tuberculosis*	SCO:bacA	Single cross over mutant
*M. tuberculosis*	SCO:cobIJ	Single cross over mutant
*M. tuberculosis*	∆*bacA*	Deletion mutant
*M. tuberculosis*	∆	Deletion mutant
*M. tuberculosis*	H37Rv::attB + rsB12	Wild type complemented with reporter system
*M. tuberculosis*	∆*bacA::attB* + rsB12	Mutant complemented with reporter system
*M. tuberculosis*	∆*cobIJ::attB* + rsB12	Mutant complemented with reporter system

### Bacterial cultures

#### *E. coli* Top10

Bacteria were cultured at 37 °C for 18–20 h in liquid or solid Luria–Bertani broth. Where necessary, the media were supplemented with antibiotics or other supplements at the following concentrations: kanamycin (Bioshop) 50 μg/ml; ampicillin (Bioshop) 100 μg/ml, X-gal 40 μg/ml (BioShop), sucrose 2% (Sigma Aldrich) at 37 °C.

#### Non-tuberculous and tuberculous mycobacteria

Where necessary, media were supplemented with 10% oleic acid albumin dextrose catalase growth supplement (OADC) (Becton–Dickinson), 0.05% Tween 80 (Sigma), tyloxapol 0.015% (Sigma-Aldrich), cobalt chloride 12 μg/ml (Sigma Aldrich), kanamycin 25 μg/ml (BioShop); X-gal 40 μg/ml (BioShop), sucrose 2% (Sigma Aldrich), vitamin B12 (adenosylcobalamin) 10 μg/ml (Sigma Aldrich), uroporfirynogen III octamethyl ester 1 μg/ml (Sigma Aldrich) (dissolved in 25% DMSO), OAD (0.05% Oleic Acid, 5% bovine serum albumin, fraction V, 2% glucose, and 0.85% NaCl**)**. All cultures were started at OD_600_ = 0.1.

Unless stated otherwise, cultures and seed cultures of non-tuberculous mycobacteria and *M. tuberculosis* were cultivated in 7H9 broth supplemented with OADC, Tween 80, and cobalt chloride. Cultures of non-tuberculous mycobacteria were started at OD600 = 0.05 and carried out until they reached OD600 = 1. *M. tuberculosis* cultures were started at OD600 = 0.1. For seeding, the appropriate amount of logarithmic phase culture (OD600 = 0.8) was spinned down, washed in fresh medium, spinned again, and re-suspended in fresh medium.

For testing vitamin B12 concentration, non-tuberculous mycobacteria were cultured in 7H9 broth supplemented with OADC, Tween 80, and cobalt chloride until they reached OD600 = 1. Starved cultures of *M. smegmatis* were cultured as described previously^17^.

We tested several types of cultures of *M. tuberculosis* for vitamin B12 concentration. Logarithmic phase cultures of *M. tuberculosis* were grown in 7H9 medium supplemented with OADC, Tween80, and cobalt chloride until they reached OD600 = 0.8. Stationary phase cultures were carried out in the same medium, and they were collected after 15 days of culture. Acidified cultures (pH 5.7) were carried out in 7H9 broth, supplemented with 5% bovine serum albumin, fraction V, 2% glucose, and 0.85% NaCl, Tween 80, and cobalt chloride. They were collected after 1 week of culture. Starved cultures were carried out in 7H9 broth supplemented with Tween 80, 0.5% glycerol, and cobalt chloride. They were collected after one week of culture. Hypoxic cultures were carried out as previously described^37^. In brief, starter cultures of *M. tuberculosis* grown on Dubos medium supplemented with OAD and cobalt chloride were tightly locked in flasks with 0.25 headspace ratio and cultured at 37 °C on a shaker for six weeks. Catalase is not recommended for use in hypoxia experiments because it influences redox balance, and redox stress is an important stress factor of hypoxia^38^. Methylene blue was added to control cultures as an indicator of oxygen depletion. After this time, the flasks were opened, cultures were spinned down and washed three times with 7H9 medium. A sample of the culture was plated as viability control, while the rest was lyzed. For persister cultures, we used a medium deprived of K+, as previously described^16^. Here, starter cultures grown on Sauton medium were spinned down and re-suspended in Sauton medium deficient in K+ supplemented with cobalt chloride. After two weeks, rifampicin was added to cultures at 5 μg ml^−1^ and the culture continued for the next four weeks at 37 °C.

### Cloning strategy

**Figure 6:**
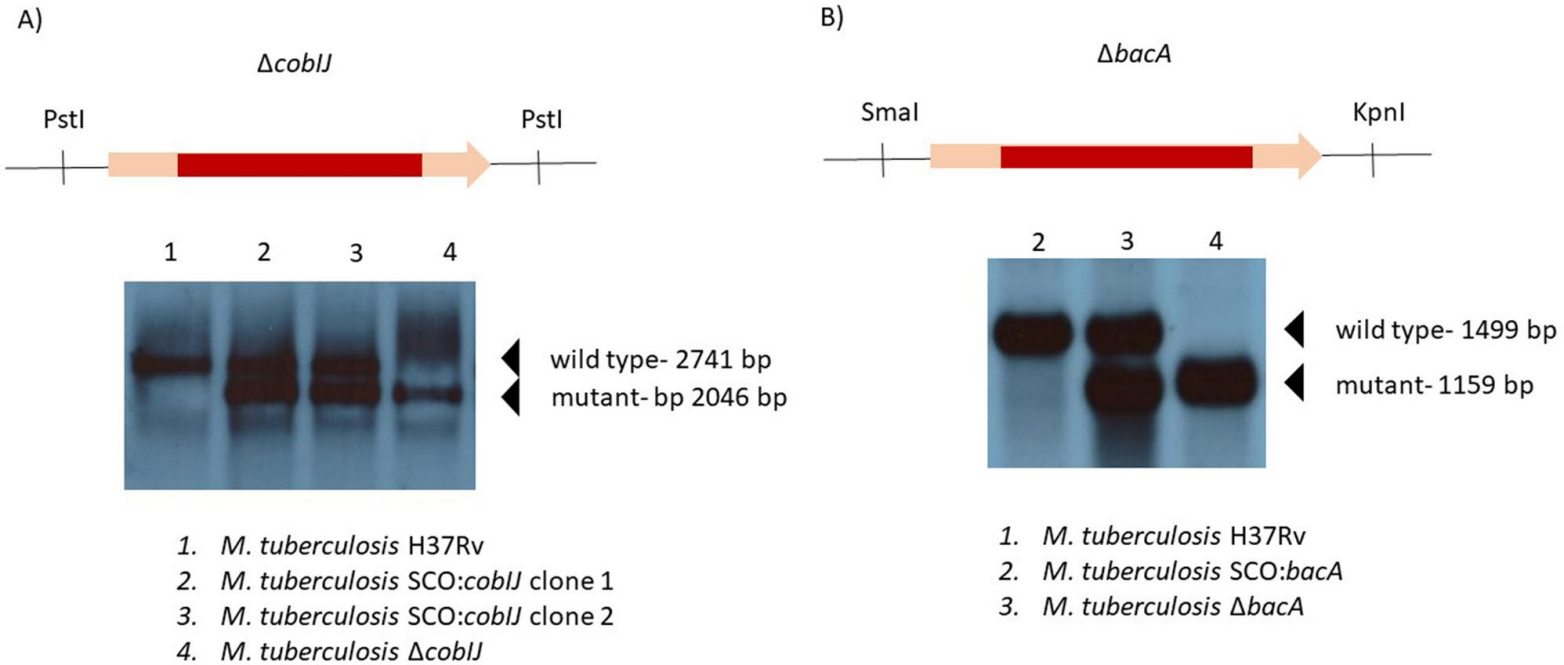
Southern blots confirming deletion of genes *cobIJ* and *bacA* in *M. tuberculosis* H37Rv. We used a gene replacement method through homologous recombination to obtain unmarked genetic mutants with large deletions inside the genes. Single cross-over (SCO) describes an intermediate step of mutagenesis. The images are cropped, hence altered lane numbering. Full-size images can be found in the supplementary data.

All molecular cloning was performed in *E. coli* T10. Knock-out mutants of mycobacteria were obtained by the method of gene replacement through homologous recombination (Tables 5, 6; Fig. 6; Supplementary Figs. 1 and 2)^39^. Briefly, sequences flanking desired deletion were amplified by PCR. We used AccuPrime Pfx High Fidelity Polymerase (Invitrogen), and genomic DNA of *M. tuberculosis* H37Rv for the reaction. PCR products were introduced into pJET1.2 plasmid (Thermo Fisher Scientific) and sequenced. Following confirmation of cloning of proper sequence, we cut out flanking sequences using restriction enzymes, and sequentially introduced them into p2NIL plasmid, together with marker genes from pGOAL17. Plasmids were transformed into *M. tuberculosis* H37Rv thru electroporation. The cells underwent gene replacement by allelic exchange as described previously^39^. Similarly, episome plasmid containing green fluorescence protein (GFP) gene under the control of the riboswitch of *M. tuberculosis metE* gene was constructed with a similar procedure. We started with PCR amplification of products on genomic DNA of *M. tuberculosis* H37Rv and pJAM plasmid carrying *gfp*. Subsequently, we introduced sequences to pJET1.2, and we confirmed proper cloning by sequencing. Next, we used restriction digestion to cut out the sequences, and we introduced them into pMV306 episome plasmid. *M. tuberculosis* H37Rv and its derivative strains were transformed by electroporation.

**Table 5:** List of primers used in this study.

Name	Primer orientation	Primer sequence 5′
**Gene replacement**		
*bacA* first flank	Forward	CAGTACTAGGTTGGATCGGCGTGGATAAGC
	Reverse	CAAGCTTCACAGATGGCACTGATCGTCCAGG
*bacA* second flank	Forward	CAAGCTTGGCGAGCGGGTGGAAGGTACC
	Reverse	CGGTACCAATACCGCCCACCCCACC
*cobIJ* first flank	Forward	CAGTACTGCGACCCATTCTCCCGTACG
	Reverse	CAAGCTTCGTGTGGGGCGCTGTGATAG
*cobIJ* second flank	Forward	CAAGCTTGACTGGATGACACCGCAGAGCC
	Reverse	CGGTACCATCACCTGGCAGATCCGCG
**Gene complementation**		
*metE promoter region*	Forward	CGGTACCCTCGGGAACCGGCTTTAACACGG
	Reverse	CTCTAGAGGTGTTCACCGGCACCGAGTCC
Green fluorescence protein	Forward	CTCTAGAATGAGTAAAGGAGAAGAACTTTTCACTGG
	Reverse	CAAGCTTCTATTTGTATAGTTCATCCATGCCATGTG
**Southern blot**		
*bacA* probe	Forward	GCGGCGAGAACGAGACGATG
	Reverse	CGCCACCGAGTAGTTCGAGCTG
*cobIJ* probe	Forward	ATGAGCGCTCGGGGCACGC
	Reverse	TCAGTCGCTGTGGCGGCTCG
**qPCR**		
*cobG*	Forward	CGCTCGTGTGTCGGTGACGG
	Reverse	AGTGCACCAGGCCGCTGACG
*cobL*	Forward	ACGCGCGACCGTGGTGTTC
	Reverse	TCGACACGTGCGGCAGCA
*cobO*	Forward	TCGTCGCTGCCGTGTTTGC
	Reverse	GGCGTTCGGGATGGCGTT
*cobU*	Forward	ACGGTCTGCCAGTGTGCGGG
	Reverse	CCGGGAAATCGCAGTGGGC
*cobD*	Forward	TGGCGCTGTTCGGTTCCGG
	Reverse	CCAGGTGTGGGCGGTTTCTGC
*cobN*	Forward	GTGGTCAGCGGCGAGCAGAC
	Reverse	AGGGGGCGTTCGAGGATGC
*sigA*	Forward	AGAAAGCCCCGGCCAAGCG
	Reverse	GCGTCGCGGCATCAGCTTCT
**PCR confirmation of gene complementation**		
pMV306	Forward	GTGGATAACCGTATTACCGC
	Reverse	AAGGCCCAGTCTTTCGACTGAG

**Table 6:** List of plasmids used in this study.

Name	Description	Source
pJET1.2	Commercial plasmid	Thermo Fisher Scientific
pJAM + gfp	pJAM plasmid carrying green fluorescence protein gene	Institute of Medical Biology
p2NIL	Recombination vector, nonreplicating in mycobacteria	Parish and Stocker, 2000
pGOAL17		Parish and Stocker, 2000
pMV306	Mycobacterial integrating vector	Med-Immune Inc
pAM1	pJET1.2 carrying first flank of cobIJ gene	This study
pAM2	pJET1.2 carrying second flank of cobIJ gene	This study
pAM3	p2NIL carrying first flank of cobIJ gene	This study
pAM4	p2NIL carrying first and second flank of cobIJ gene	This study
pAM5	p2NIL plasmid carrying flanking sequences of deletion within cobIJ gene, and marker genes of pGOAL17 plasmid, KmR, lacZ+	This study
pAB1	pJET1.2 carrying first flank of bacA gene	This study
pAB2	pJET1.2 carrying second flank of bacA gene	This study
pAB3	p2NIL carrying first flank of bacA gene	This study
pAB4	p2NIL carrying first and second flank of bacA gene	This study
pAB5	p2NIL plasmid carrying flanking sequences of deletion within bacA gene, and marker genes of pGOAL17 plasmid, KmR, lacZ+	This study
pAM6	pJET1.2 carrying metE promoter region	This study
pAM7	pJET1.2 carrying gfp	This study
pAM8	pMV306 carrying metE promoter region	This study
pAM9	pMV306 plasmid carrying green fluorescence protein (GFP) gene under the control of the riboswitch of *M. tuberculosis* metE gene	This study

### Vitamin B12 ELISA

Bacterial cultures were spinned down, washed with fresh medium without supplements, spinned down again, and re-suspended in 0.01 M TRIS pH 7.5. The mixture was transferred to disruptor eppendorfs. Cells were disrupted twice using the MP disruptor system with the Quick prep adapter (MP Biomedicals) and 0.1 mm silica spheres (45 s, 6.0 m/s with 5 min intervals). Samples were spinned down, and lysates were transferred to new eppendorfs. As a principal, we normalized the results regarding vitamin B12 concentration to protein concentration in cell lysates. We wanted to avoid errors resulting from a different level of difficulty to disrupt mycobacterial cells of different species. Protein concentration in lysates was measured using Bradford reagent (BioShop) and estimated with a standard curve. In order to achieve a sufficient detection limit of vitamin B12, we only used the lysates that contained at least 0.5 mg of protein per ml, preferably between 1 and 2 mg of protein per ml.

Vitamin B12 ELISA (Demeditec) was performed according to manufacturer instructions. The test is based on the principle of the competitive enzyme-linked immunosorbent assay. The surface of a microtiter plate was covered with an antibody directed against vitamin B12 by the manufacturer. Samples and standards were mixed with a vitamin B12-peroxidase conjugate in the wells of the microtiter plate. Both enzyme-labeled and free vitamin B12 competed for the antibody binding sites. After one hour of incubation at room temperature, the wells were washed to remove the unbound material. A substrate solution was added, resulting in the development of a blue color. The color development was inhibited by the addition of a stop solution, and the color turned yellow. The yellow color was measured photometrically at 450 nm. The concentration of vitamin B12 was indirectly proportional to the color intensity of the test sample.

For each condition, we analyzed lysates from three independent cultures. Each sample was analyzed in duplicate wells, as recommended by the producer of the immunoassay. The minimum detection level of vitamin B12 was settled at 1 ng/ml based on our previous observations^17^, and all samples below this level were considered negative for vitamin B12. For purposes of enabling comparison with the results obtained from other species of bacteria, our results were also normalized to ml of culture.

### Flow cytometer analysis

Samples of cultures were analyzed on the flow cytometer Guava EasyCyte Flow Cytometer with High Power Blue Laser (Merck) suitable for detection of bacteria. Unstained control samples were diluted to reach a concentration of 400–800 cells/μl. Cell suspensions were first run through the flow cytometer to set a population gate around the bacteria by using the forward-scatter versus side-scatter parameters. Next, the voltages in the green fluorescence channel were adjusted so that the fluorescence histogram of the unstained bacteria appeared within the first compartments of the logarithmic scale of fluorescence. Ten thousand events were collected at a set standard low event rate. We used Guava software to analyze the acquired data. For each strain, we analyzed data from three cultures.

### qPCR

Bacterial cultures were spinned down, re-suspended in water, and three volumes of TriReagent was added (Bioshop). The mixture was transferred to disruptor eppendorfs. Cells were disrupted twice using the MP disruptor system with the Quick prep adapter (MP Biomedicals) and 0.1 mm silica spheres (45 s, 6.0 m/s with 5 min intervals). Samples were spinned down, and the supernatant was transferred to new eppendorfs. One volume of chloroform was added, samples were vigorously mixed and spinned down. The top phase was transferred to new eppendorfs and precipitated with 1 volume of isopropanol and 1/10 volume of sodium acetate. Following precipitation, samples were re-suspended in water and digested with Turbo DNase I (Invitrogen by Thermo Fisher Scientific) following the manufacturer's instructions. The RNA quantity was assessed using a NanoDrop 2000 spectrophotometer (Thermo Fisher Scientific). cDNA was obtained using SuperScript III First-Strand Synthesis Super Mix kit with random hexamers (Invitrogen). qPCR was performed using SG qPCR Master Mix (2×), plus ROX Solution (Eurx), and synthetic primers (Table 5) on a 7900HT real-time PCR system (Applied Biosystems). Real-time PCR conditions were as follows: initial activation at 95 °C for 10 min, followed by 40 cycles at 94 °C for 15 s (denaturation), 62 °C for 30 s (annealing), 72 °C for 30 s (extension). The melting curve analysis was performed at the end of each qPCR reaction to verify a single, specific product was generated. The threshold cycle (CT) value for each studied gene was normalized to the expression of msmeg_2758 (*sigA*) (ΔCT) and converted to linear form (2 − ΔC_T_). The RNA samples for each strain were isolated from three independently grown cultures. Each sample for qPCR was run in triplicate.

### RNA Seq

Raw RNA Seq reads were downloaded from European Nucleotide Archive Database (ENA). We analyzed gene expression of *M. abscessus* subsp*. abscessus* grown in 7H9 medium supplemented with OADC^40^, *M. smegmatis* grown in 7H9 medium with glucose^41^, and three experiments performed with *M. tuberculosis* H37Rv grown in 7H9 broth supplemented with OADC^20^, in a medium supplemented with cholesterol as a sole carbon source^21^ and in human THP-1 derived macrophages three days post-infection. Each experiment contained data for three replicates. Raw sequences were uploaded and processed with Geneious Prime 2021 (Biomatters, New Zealand). Reads were mapped to *M. tuberculosis* H37Rv accession number NC_000962 using Bowtie2 Geneious plug-in^42^. Gene expression analysis, through estimation of transcripts per kilobase million (TPM), was performed with Geneious.

### Identification of loci in the whole genome sequencing data based on the genome annotation

We identified genes involved in vitamin B12 metabolism in the following strains: *M. tuberculosis* H37Rv (NC_000962), *M. abscessus* subsp*. abscessus* ATCC19977 (NC_010397), *M. abscessus* subsp*. bolletii* FLAC 003 (CP014950), *M. conspicuum* JCM 14738 (GCA_010730195), *M. fortuitum* CT6 (CP011269), *M. gastri* DSM 43505 (LQOX1000000), *M. gordonae* 24T (CP059165), *M. innocens* MK13 (LS999933), *M. kansasii* ATCC 12478 (GCA_000157895.1), *M. persicum* H48 (GCA_002705835), *M. phlei* CCUG 21000 (GCA_001582015), *M. porcinum* ACS 3670 (NZ_MBDY01000007.1), *M. terrae* NCTC 10856 (GCA_900187145), *M. xenopi* RIVM700366 (NZ_AJFI01000095.1), *M. szulgai* DSM 44166 (NZ_LQPW01000016.1) and *M. smegmatis* mc^2^ 155 (CP009494). We screened the following databases: National Center of Biotechnology Information, Nucleotide and Protein (NCBI), Mycobrowser, STRING, UniProt, and we manually screened the sequences thru Geneious Prime (Biomatters, New Zealand).

### Statistical analysis

Statistical analysis was performed with Develve Statistical Software, with paired t-test. The level of statistical significance was *p* < 0.05. All results are reported as the means ± SD unless otherwise stated.

## Supplementary Information

Supplementary Figures.
