# Supplementary material for: Cobalamin is present in cells of non-tuberculous mycobacteria, but not in Mycobacterium tuberculosis
Source: Sci Rep. 2021 Jun 10;11:12267. doi: 10.1038/s41598-021-91430-w (PMC8192938; doi:10.1038/s41598-021-91430-w)
Supplement: Supplementary file 1 — Supplementary Figures. [file 41598_2021_91430_MOESM1_ESM.pdf]

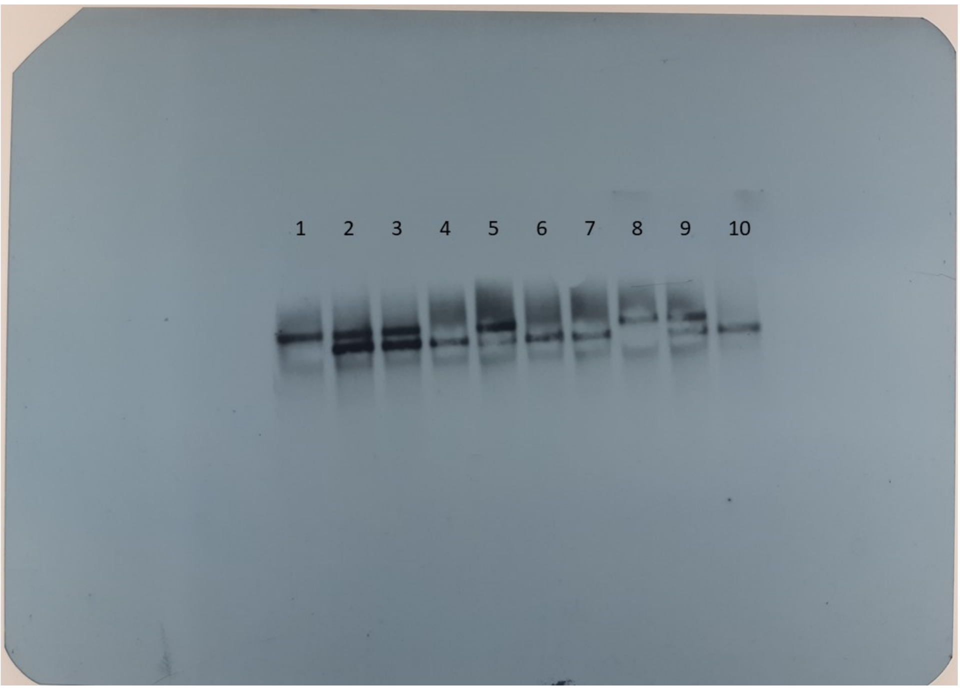

Supplementary Fig. 1. Full-length Southern blot confirming deletion of *cobIJ* in *M. tuberculosis* H37Rv. We used a gene replacement method through homologous recombination to obtain unmarked genetic mutants with large deletions inside the genes. Single cross-over (SCO) describes an intermediate step of mutagenesis. 1) *M. tuberculosis* H37Rv, 2) *M. tuberculosis* SCO clone 1, 3) *M. tuberculosis* SCO clone 2, 4) *M. tuberculosis* Δ*cobIJ* clone 1 5) *M. tuberculosis* Δ*cobIJ*- SCO, erroneous gene replacement mutant, 6) *M. tuberculosis* Δ*cobIJ* clone 2, 7) *M. tuberculosis* Δ*cobIJ* clone 3, 8) *M. tuberculosis* Δ*bacA*, 9) *M. tuberculosis* Δ*cobIJ*- SCO in Δ*bacA* bacground, 10) *M. tuberculosis* Δ*bacA*/Δ*cobIJ* clone 1.

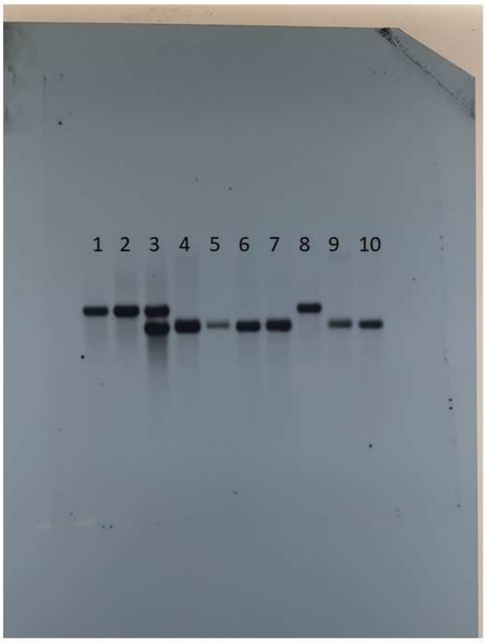

Supplementary Fig. 2. Full-length Southern blot confirming deletion of *bacA* in *M. tuberculosis* H37Rv. We used a gene replacement method through homologous recombination to obtain unmarked genetic mutants with large deletions inside the genes. Single cross-over (SCO) describes an intermediate step of mutagenesis. 1) *M. tuberculosis* H37Rv, 2) *M. tuberculosis* H37Rv, 3) *M. tuberculosis* SCO , 4) *M. tuberculosis* Δ*bacA* clone 1 5) *M. tuberculosis* Δ*bacA* clone 2, 6) *M. tuberculosis* Δ*bacA* clone 3, 7) *M. tuberculosis* Δ*bacA* clone 4, 8) wild type revertant clone, 9) *M. tuberculosis* Δ*bacA* clone 5, 10) *M. tuberculosis* Δ*bacA* clone 6.
